# *ARMCX3* Regulates Breast Cancer Progression and Cisplatin Responsiveness Through p53-Related Signaling

**DOI:** 10.3390/biology15201853

**Published:** 2026-10-15

**Authors:** Xinyan Deng, Haixiang Ma, Jiajie Zhang, Xiyang Huang, Minghong Cai, Fanghui Ge, Hong Zhang, Jiandong Wang

**Affiliations:** 1 Sichuan Provincial Engineering Laboratory for Prevention and Control Technology of Veterinary Drug Residue in Animal-Origin Food, School of Laboratory Medicine, Chengdu Medical College, Chengdu 610500, China; 17828347540@163.com (X.D.); 13308012660@163.com (H.M.); 17358697721@163.com (J.Z.); 17311251676@163.com (X.H.); 2 Experiment Teaching Demonstration Center of Laboratory Medicine, School of Laboratory Medicine, Chengdu Medical College, Chengdu 610500, China; 3 School of Bioscience and Technology, Chengdu Medical College, Chengdu 610500, China; 15882474900@163.com

**Keywords:** *ARMCX3*, breast cancer, p53 signaling, cisplatin sensitivity

## Abstract

Despite substantial advances in diagnosis and treatment, breast malignancy continues to cause considerable morbidity and mortality. Cisplatin has demonstrated therapeutic activity in selected patients with advanced breast cancer, but its clinical use may be limited by treatment resistance. Although *ARMCX3* is implicated in other cancers, its role and underlying mechanisms in breast cancer remain unclear. We investigated *ARMCX3* functions using depletion and rescue experiments, and *ARMCX3* depletion reduced cell growth, migration, and invasion and increased cisplatin sensitivity in vitro, while re-expression rescued these phenotypes. Transcriptomic analysis revealed enrichment of the p53 pathway following *ARMCX3* depletion. PFT-α treatment suppressed the protein level of p53 and attenuated the effects of *ARMCX3* knockdown on cell proliferation, migration, invasion, cell-cycle arrest, and cisplatin response. *ARMCX3* knockdown was also associated with altered DNA damage response signaling, enhanced cisplatin-induced apoptosis and suppressed tumor growth in vivo, and all these effects were attenuated by PFT-α treatment. These findings suggest that p53-associated signaling contributes to the effects of *ARMCX3* depletion on breast cancer progression and cisplatin responsiveness. These results may help inform future studies exploring *ARMCX3*-associated signaling in the context of cisplatin response.

## Simple Summary

Breast cancer remains a leading cause of cancer-related death among women, and cisplatin resistance limits chemotherapy efficacy in patients with advanced breast cancer. Here, we investigated the role of *ARMCX3* in breast cancer progression and cisplatin responsiveness. *ARMCX3* depletion reduced cell growth and suppressed migratory and invasive capacities while increasing sensitivity to cisplatin treatment in vitro, although re-expression of *ARMCX3* rescued these phenotypes. Transcriptomic and molecular analyses further suggested that *ARMCX3* depletion was associated with increased p53-related signaling, altered DNA damage response signaling and enhanced apoptosis following cisplatin treatment. Overall, our findings provide further insight into the role of *ARMCX3* in breast cancer progression and cisplatin responsiveness and support further investigation of *ARMCX3*-associated signaling in the context of cisplatin response.

## 1. Introduction

Breast malignancies impose a substantial disease burden on the global female population and account for a considerable proportion of cancer-associated mortality [1]. Worldwide estimates for 2022 documented nearly 2.3 million newly diagnosed cases and approximately 670,000 related deaths [2]. Despite substantial advances in surgery, endocrine therapy, targeted therapy, and immunotherapy, chemotherapy remains an essential component of treatment for patients with advanced or metastatic breast cancer [3]. Among various chemotherapeutic agents, cisplatin, a platinum-based compound, exerts its antitumor effects primarily through the formation of DNA crosslinks, induction of DNA damage, and activation of apoptosis-related pathways. Cisplatin has demonstrated therapeutic activity in selected breast cancer subtypes, particularly in triple-negative breast cancer; however, the development of acquired resistance substantially limits its clinical efficacy [4,5,6]. Increasing evidence indicates that dysregulation of DNA damage responses and apoptotic signaling contributes to platinum resistance, yet the molecular mechanisms underlying these processes remain incompletely understood [7,8,9]. Therefore, identifying novel regulators of breast cancer progression and molecular determinants that influence cellular responses to cisplatin may provide new opportunities for improving therapeutic efficacy. *ARMCX3* (Armadillo Repeat-Containing X-Linked Protein 3) is a highly conserved member of the *ARMCX* family that localizes predominantly to the outer mitochondrial membrane, where it participates in mitochondrial transport, dynamics, and cellular homeostasis [10,11,12]. Previous studies have implicated *ARMCX3* in multiple biological processes, including neural development, Wnt/β-catenin signaling, and metabolic regulation [13,14]. Emerging evidence also suggests that *ARMCX3* contributes to tumor progression. For example, *ARMCX3* is upregulated in hepatocellular carcinoma, and its depletion suppresses tumor cell proliferation and migration [15]. However, the role of *ARMCX3* in breast cancer and the molecular mechanisms underlying its effects on tumor cell behavior and cisplatin responsiveness remain poorly understood.

DNA damage responses are critical for maintaining genomic stability and determining cell fate following genotoxic stress. Among the key mediators of these responses, p53 is a central tumor suppressor that coordinates cell-cycle arrest, DNA repair, apoptosis, and cellular responses to genotoxic stress [16,17]. Loss of p53 function, most commonly caused by *TP53* mutation, promotes tumor progression and therapeutic resistance [18,19,20]. However, the ARMCX3 protein, which is predominantly localized to the outer mitochondrial membrane, participates in the regulation of DNA damage responses or p53 signaling in breast cancer and has not been systematically investigated. Thus, in the present study, we sought to determine whether *ARMCX3* functionally influences breast cancer progression and cellular responses to cisplatin through these signaling networks.

We first investigated the expression pattern of *ARMCX3* in breast cancer using publicly available datasets and evaluated its association with clinical outcomes. We then established *ARMCX3* knockdown breast cancer cell models to investigate its role in tumor-associated behaviors and cisplatin responsiveness. To assess the on-target specificity of shRNA-mediated *ARMCX3* depletion, an shRNA-resistant *ARMCX3* construct was transfected into *ARMCX3*-deficient cells for rescue experiments. Subsequently, RNA sequencing was performed to characterize the transcriptional alterations associated with *ARMCX3* depletion and to identify potential signaling pathways involved in its biological functions. Based on the transcriptomic findings, we further examined p53-associated signaling, cell-cycle regulation, apoptosis, and DNA damage response signaling using molecular, cellular, and pharmacological approaches. Xenograft experiments were additionally performed to evaluate the effect of *ARMCX3* depletion on tumor growth in vivo. Collectively, this study aimed to investigate the signaling mechanisms associated with the effects of *ARMCX3* depletion on breast cancer progression and cisplatin responsiveness, with particular emphasis on p53-associated signaling. These findings provide further insight into the biological and molecular effects of *ARMCX3* depletion in breast cancer and support further investigation of its potential relevance to cisplatin responsiveness.

## 2. Materials and Methods

### 2.1. Public Database Analysis

*ARMCX3* mRNA expression was analyzed using the TCGA–GTEx breast cancer RNA-sequencing dataset obtained from the UCSC Xena database. Expression differences among TCGA primary breast tumors, TCGA adjacent normal tissues, and GTEx normal mammary tissues were compared. ARMCX3 protein expression in primary breast tumors and normal breast tissues was subsequently analyzed using CPTAC proteomic data obtained through the UALCAN web resource. ARMCX3 immunohistochemical staining in nonmalignant mammary and breast carcinoma tissues was examined using data from the Human Protein Atlas (HPA) [21]. The association between ARMCX3 expression and overall survival was further evaluated using the HPA Pathology Atlas. Patients were stratified into relatively high- and low-expression groups according to the database-generated optimal cutoff, followed by Kaplan–Meier survival analysis.

### 2.2. Cell Culture

The human breast cancer cell lines MCF-7 and MDA-MB-231, purchased from the Cell Bank of the Chinese Academy of Sciences (Shanghai, China), were cultured in high-glucose DMEM medium (Procell, Wuhan, China, PM150210), supplemented with 10% fetal bovine serum (Vivacell, Shanghai, China, C04001-500) and 1% penicillin–streptomycin solution (Biosharp, Beijing, China, BL505A). MCF-7 cells represent the luminal A subtype of breast cancer and harbor wild-type *TP53*, whereas MDA-MB-231 cells represent the triple-negative/basal-like subtype and carry the *TP53* R280K missense mutation. Cells were maintained at 37 °C, 5% CO_2_. Lentiviral vectors encoding *ARMCX3* short hairpin RNA (shARMCX3) and the corresponding negative control (shControl) were designed, constructed, and packaged by HanHeng Biotechnology (Shanghai, China). The correct targeting sequence of shARMCX3 was the 19-nt sense strand 5′-GGC TTA AAG TAT ACA TGA A-3′. The validated shARMCX3 oligonucleotides used for vector construction were: Top strand: 5′-GAT CCG GCT TAA AGT ATA CAT GAA CTC GAG TTC ATG TAT ACT TTA AGC CTT TTT TG-3′; Bottom strand: 5′-AAT TCA AAA AAG GCT TAA AGT ATA CAT GAA CTC GAG TTC ATG TAT ACT TTA AGC CG-3′. All cells were incubated with the virus at 37 °C for 24 h, after which the medium was replaced with fresh DMEM containing 5 μg/mL puromycin (Solarbio, Beijing, China, Cat#P8230). After 3 days of antibiotic selection, viable cells were expanded in 6-well plates and transferred into T25 flasks for subsequent experiments.

### 2.3. ARMCX3 Re-Expression

To perform *ARMCX3* rescue experiments, stable *ARMCX3* knockdown MCF-7 and MDA-MB-231 cells were transiently transfected with the shRNA-resistant human *ARMCX3* expression plasmid h-*ARMCX3* (MUT)-3 × FLAG/pcDNA3.1-EGFP using LipoFiter 3.0 Transfection Reagent (Hanbio Biotechnology Co., Ltd., Shanghai, China). The shRNA-resistant *ARMCX3* construct contained a 3 × FLAG tag and was introduced into *ARMCX3*-depleted cells to restore *ARMCX3* expression. Cells transfected with the corresponding pcDNA3.1-EGFP empty vector were used as vector controls. Cells were seeded 20–24 h before transfection and transfected at approximately 70% confluence. Plasmid DNA and LipoFiter 3.0 were prepared and mixed according to the manufacturer’s instructions. Following transfection, cells were maintained under standard culture conditions and subsequently subjected to Western blotting, cell proliferation, migration, invasion, and cisplatin-sensitivity assays.

### 2.4. Bioinformatics

The transcriptomic dataset produced in the present work is publicly available through the Gene Expression Omnibus repository with the identifier GSE308702. Raw gene-level count data were analyzed in R (v4.3.1). Count normalization and statistical comparison between experimental groups were carried out with DESeq2. Following the criteria reported previously, genes showing an absolute log_2_ fold change greater than 0.585, corresponding to an FC above 1.5 or below 0.67, together with an FDR below 0.05, were regarded as differentially expressed. Positive and negative log_2_ fold-change values were used to classify the identified genes as upregulated and downregulated, respectively. The overall distribution of differential expression was illustrated with ggplot2 (v3.4.4), whereas expression patterns across samples were displayed using pheatmap (v1.0.12). For graphical presentation of individual gene abundance, normalized count values obtained from DESeq2 were converted using the formula log_2_ (count + 1).

Functional characterization of the differentially expressed genes was carried out in R using clusterProfiler (v4.4.4). Enrichment of biological functions and signaling pathways was examined against the human Gene Ontology and KEGG annotation databases. A hypergeometric model was used to calculate enrichment, and the resulting *p* values were corrected for multiple testing by the Benjamini–Hochberg procedure. Enriched categories with an FDR-adjusted value of less than 0.05 were considered statistically meaningful. Results were displayed as bubble plots, with the enrichment factor, mapped gene number, and adjusted Q value used to describe the magnitude and significance of enrichment. Gene set enrichment analysis (GSEA) was additionally performed using the gene-expression matrix to evaluate coordinated changes in predefined gene sets between shControl and shARMCX3 cells. Genes were ranked using the Signal2Noise metric, and enrichment scores were calculated across the ranked gene list. GSEA was performed separately for MCF-7 and MDA-MB-231 cells, and enrichment plots were generated for representative p53-related gene sets.

To assess the overall distribution of gene expression across samples, violin plots were generated using the ggplot2 package (v3.4.4) in R (v4.3.1), based on the log_2_ (FPKM + 1) values of all detected genes in each sample. Pearson correlation coefficients were calculated from the FPKM expression matrix to evaluate sample consistency and biological reproducibility. Sample correlation heatmaps were generated using the pheatmap package (v1.0.12), with pairwise correlation coefficients displayed in the matrix. Principal component analysis (PCA) was performed using the prcomp function in base R, and PCA plots were generated using ggplot2 to evaluate sample clustering and potential batch effects.

Cellular RNA was isolated with TRIzol reagent supplied by Tiangen Biotech (Beijing, China; DP424). RNA quality was subsequently assessed by examining sample purity and integrity, and only qualified preparations were subjected to library construction. Messenger RNA libraries were prepared using the Hieff NGS Ultima Dual-mode mRNA Library Prep Kit for Illumina (Yeasen Biotechnology, Shanghai, China; 12301ES96) according to the protocol reported previously [22].

Poly(A)-containing transcripts were isolated from total RNA by capture with oligo(dT)-coated magnetic particles and then sheared to an appropriate length. The fragmented RNA served as the template for synthesis of the first cDNA strand using random hexamers. The complementary strand was subsequently generated with dNTPs; when a strand-specific protocol was applied, dTTP was replaced by dUTP at this step. After purification, the cDNA products underwent end repair, 3′ A-tailing, and attachment of sequencing adapters. Fragments of the required length were selected before library amplification. For strand-specific libraries, the dUTP-containing strand was enzymatically removed before PCR, thereby retaining information on transcript orientation. Preliminary library quantification was carried out with a Qubit 3.0 fluorometer (Invitrogen, Carlsbad, CA, USA), followed by qPCR-based determination of the amplifiable library concentration. Libraries that met the quality-control requirements were subjected to 150-bp paired-end sequencing on the NovaSeq 6000 system (Illumina, San Diego, CA, USA).

### 2.5. Protein Interaction Analysis

The protein–protein interaction (PPI) network was constructed using the Search Tool for the Retrieval of Interacting Genes/Proteins (STRING) database (https://string-db.org) by importing the differentially expressed genes (DEGs) to identify potential protein interactions. The resulting interaction data were then imported into Cytoscape (v3.7.1) (https://cytoscape.org) for visualization and topological analysis of the network. Core regulatory genes (hub genes) were identified using the CytoHubba plugin within Cytoscape, employing three distinct topological algorithms-Maximal Clique Centrality (MCC), Maximum Neighborhood Component (MNC), and Degree to prioritize key nodes that may play critical roles in disease mechanisms.

### 2.6. Reagents

Cell culture and experimental procedures were conducted using Dulbecco’s modified Eagle’s medium (DMEM; Procell, Wuhan, China; PM150210), fetal bovine serum (Vivacell, Shanghai, China; C04001-500), penicillin–streptomycin solution (Biosharp, Beijing, China; BL505A), and puromycin (Solarbio, Beijing, China; P8230). Protein extraction and immunoblotting were performed with RIPA lysis buffer (Beyotime, Shanghai, China), protease inhibitor cocktail (CWBIO, Beijing, China; CW2200S), phosphatase inhibitor cocktail (CWBIO; CW2383S), a bicinchoninic acid protein assay kit (Thermo Fisher Scientific, Waltham, MA, USA; 23225), and an enhanced chemiluminescence substrate (Millipore, Billerica, MA, USA). Additional reagents included Cell Counting Kit-8 reagent (Biosharp, Beijing, China; BS350A), crystal violet staining solution (Beyotime; C0121), Matrigel basement membrane matrix (Corning, Corning, NY, USA; 356234), propidium iodide/RNase A staining solution (KeyGEN BioTECH, Nanjing, China; KGA9101-50), a comet assay kit (KeyGEN BioTECH, Nanjing, China; KGA1302-100), paraformaldehyde (Servicebio, Wuhan, China; G1101-500ML), and 3,3′-diaminobenzidine staining solution (Zhongshan Golden Bridge Biotechnology, Beijing, China; ZLI-9017).

### 2.7. Western Blotting

At the end of treatment, total cellular protein was extracted using RIPA buffer supplemented with a protease inhibitor cocktail (CWBIO, Beijing, China; CW2200S) and a phosphatase inhibitor cocktail (CWBIO; CW2383S). Protein content was quantified with a bicinchoninic acid assay kit (Thermo Fisher Scientific, Waltham, MA, USA; 23225). Equal amounts of protein were denatured, separated by SDS–polyacrylamide gel electrophoresis, and electrotransferred onto polyvinylidene difluoride membranes. Nonspecific binding sites were blocked by incubating the membranes in 5% nonfat dry milk.

The membranes were then maintained at 4 °C overnight with antibodies against β-actin (1:10,000; Abways, Shanghai, China), p53 (1:1000; Abways), phospho-p53 (1:1000; Abways), p21/CDKN1A (1:1000; Abways), CDK2 (1:1000; Abways), Cyclin E1 (1:1000; Abways), Flag tag (1:1000; Abways), ERK1/2 (1:2000; Proteintech, Wuhan, China), ARMCX3 (1:1000, Proteintech), phospho-ERK1/2 (1:2000; Cell Signaling Technology, Danvers, MA, USA), ATM (1:1000, Cell Signaling Technology), phospho-ATM (1:1000; Cell Signaling Technology), Histone H2AX (1:1000, Abcam, Cambridge, UK), phospho-histone H2AX (1:1000; Abcam), DNA-PKcs (1:1000, Abcam), phospho-DNA-PKcs (1:5000; Abcam), E-cadherin (1:1000; Abmart, Shanghai, China), N-cadherin (1:1000; Abmart), and vimentin (1:1000; Abmart).

After removal of unbound primary antibodies by washing, the membranes were exposed to the appropriate secondary antibodies (1:10,000; Abways) for 1 h at 37 °C. Immunoreactive signals were visualized using an enhanced chemiluminescence substrate supplied by Millipore (Billerica, MA, USA). Each immunoblotting experiment was independently repeated no fewer than three times. All primary antibodies used in this study were commercially available and validated antibodies obtained from reputable suppliers. Detailed information regarding antibody source, catalog number, and dilution ratio is provided in Supplementary Table S1. The specificity of antibodies was assessed based on the expected molecular weight of target proteins and the reproducible changes in protein expression under the corresponding experimental conditions. Phospho-specific antibodies, including antibodies against γ-H2AX, p-ATM, p-DNA-PKcs, p-ERK, and p-p53, have been previously validated and widely used in published studies.

### 2.8. Cell Viability Assay

Exponentially growing cells were collected and dispensed into 96-well plates at 5 × 10^4^ cells/mL. After a 24-h attachment period under standard culture conditions (37 °C and 5% CO_2_), the indicated treatments were administered, and incubation was continued for 24, 48, or 72 h. At each designated endpoint, the spent medium was replaced with 100 μL of fresh culture medium supplemented with 10 μL of CCK-8 solution (Biosharp, Beijing, China; BS350A). Cell-free wells containing the same reaction mixture were included for background correction. Following a 2 h reaction at 37 °C protected from light, optical density was recorded at 450 nm with a microplate spectrophotometer.

### 2.9. Transwell Cell Migration Assay

Cell migration and invasion were assessed using 24-well Transwell chambers equipped with 8 μm-pore uncoated polycarbonate membranes. For the invasion assay, the upper surface of each membrane was coated with Matrigel. Briefly, Matrigel was thawed at 4 °C overnight, diluted 1:8 (*v*/*v*) with serum-free basal medium, and 50 μL of the diluted Matrigel solution was applied to each membrane. The coated chambers were incubated at 37 °C for 4–6 h to allow Matrigel polymerization, after which excess liquid was removed.

Cells in the logarithmic growth phase were treated with the indicated compounds according to the experimental design. MCF-7 cells were treated with PFT-α (20 μM) or trametinib (0.5 μM) for 48 h, whereas MDA-MB-231 cells were treated with PFT-α (30 μM) or trametinib (10 nM) for 48 h. After treatment, the cells were digested with 0.25% trypsin-EDTA, centrifuged at 1000× *g* for 5 min, and resuspended in serum-free basal medium at a final density of 1 × 10^5^ cells/mL. A total of 200 μL of cell suspension was added to the upper chamber, and 600 μL of complete medium containing 10% fetal bovine serum was added to the lower chamber as a chemoattractant. The chambers were incubated at 37 °C with 5% CO_2_ for 24 h.

After incubation, the medium in the upper chamber was removed, and non-migrated or non-invaded cells remaining on the upper surface of the membrane were removed using a sterile cotton swab. Cells attached to the lower surface of the membrane were fixed with 4% paraformaldehyde for 20 min at room temperature and stained with 0.1% (*w*/*v*) crystal violet solution (Beyotime, Shanghai, China, C0121) for 15 min. The membranes were washed with running water, air-dried at room temperature, and five randomly selected fields per membrane were imaged using an inverted light microscope (Olympus, Tokyo, Japan) at 100× magnification. Migrated or invaded cells were counted manually or quantified using ImageJ (v1.54p) software (National Institutes of Health, Bethesda, MD, USA). Each condition was tested in triplicate wells, and all assays were independently repeated at least three times.

### 2.10. Cell Cycle Assay

One milliliter of the cell suspension was collected and centrifuged at 300× *g* for 5 min. The supernatant was discarded, and 500 μL of pre-chilled 70% ethanol was added to the cell pellet. The cells were gently resuspended by pipetting and fixed at −20 °C overnight. Before staining, the fixed cells were pelleted by centrifugation at 1000× *g* for 5 min at 4 °C and washed twice with ice-cold PBS to remove residual ethanol. The cell pellet was resuspended in 500 μL of PBS containing 100 μg/mL RNase A (KeyGEN BioTECH, Nanjing, China; KGA9101-50) and incubated at 37 °C for 30 min in the dark to remove intracellular RNA. Subsequently, PI (KeyGEN BioTECH, Nanjing, China; KGA9101-50) was added to a final concentration of 50 μg/mL, and the samples were incubated for an additional 30 min at 4 °C in the dark. After staining, the samples were analyzed by flow cytometry, and PI fluorescence was detected following excitation at 488 nm.

### 2.11. Detection of Cell Apoptosis Using Annexin V-PE/7-AAD Double Staining

MCF-7 and MDA-MB-231 cells were treated with cisplatin, PFT-α, trametinib, or the indicated combinations according to the experimental design. MCF-7 cells were treated with cisplatin (10 μM), PFT-α (20 μM), or trametinib (0.5 μM), whereas MDA-MB-231 cells were treated with cisplatin (20 μM), PFT-α (30 μM), or trametinib (10 nM). Cisplatin and PFT-α were applied simultaneously and maintained for 24 h. For the combination treatment with cisplatin and trametinib, cells were first treated with trametinib for 24 h, followed by the addition of cisplatin, and were subsequently incubated for an additional 24 h. After treatment, cells were harvested using EDTA-free trypsin, washed twice with cold PBS, and centrifuged at 300 × *g* for 5 min. Approximately 1–5 × 10^5^ cells were collected and resuspended in 500 μL of binding buffer to generate a single-cell suspension. Cells were stained with 1 μL of Annexin V-PE and 5 μL of 7-AAD according to the manufacturer’s instructions. After gentle mixing, the samples were incubated for 5–10 min at room temperature in the dark. The stained cells were analyzed by flow cytometry. Annexin V-PE fluorescence was detected through the PE channel (FL2; excitation wavelength, 488 nm; emission wavelength, 578 nm), whereas 7-AAD fluorescence was used to discriminate non-viable cells.

### 2.12. Comet Assay

After adjusting the cells to the appropriate concentration, they were processed using the Comet Assay Kit (KeyGEN BioTECH, Nanjing, China; KGA1302-100). Cell pretreatment was performed according to the experimental design described in the apoptosis assay. Specifically, MCF-7 cells were treated with cisplatin (10 μM) for 24 h, whereas MDA-MB-231 cells were treated with cisplatin (20 μM) for 24 h. After treatment, cells were harvested for comet assay analysis.

The entire experimental procedure was performed according to the instructions of the Comet Assay Kit (KeyGEN BioTECH, Nanjing, China; KGA1302-100). Briefly, cells were adjusted to an appropriate concentration, embedded in low-melting-point agarose on comet assay slides, and subjected to cell lysis. DNA was then unwound under alkaline conditions and electrophoresed. Subsequently, slides were neutralized, stained with a fluorescent DNA dye, and visualized using a fluorescence microscope.

For quantitative analysis, 50 randomly selected comets were analyzed from each experimental group in each independent experiment. The olive tail moment (OTM), representing the extent of DNA damage, was calculated using Comet Assay Software (CASP, version 2.0.0.38). The experiments were performed using three independent biological replicates. For statistical analysis, the mean OTM value obtained from each biological replicate was considered as one individual data point, and statistical comparisons were performed based on biological replicates rather than individual comet measurements.

### 2.13. Establishment of a Tumor-Bearing Mouse Model

BALB/c nude mice were obtained from Jiangsu Jicui Yekang Biotechnology Co., Ltd (Nanjing, China). Following 7 days of acclimation, the animals were randomly assigned to experimental groups with six mice per group and labeled. They were maintained under specific environmental conditions, including an ambient temperature of 22 ± 2 °C, relative humidity of 40–60%, and alternating 12 h light and 12 h dark periods. MCF-7 and MDA-MB-231 cells with stable *ARMCX3* silencing were collected during exponential growth and suspended in an ice-cold 1:1 mixture of high-glucose DMEM basal medium (Procell, Wuhan, China; PM150210) and Matrigel (Corning, Corning, NY, USA; 356234). A total of 1 × 10^7^ cells in 100 μL were implanted subcutaneously into the right axillary region of each mouse.

The inoculation area was inspected at intervals of 3–4 days, and palpable xenografts generally appeared approximately 1 week after implantation. Tumor sizes were measured and recorded at 4-day intervals. Xenograft volumes were calculated using the following formula: V = L × W^2^/2, where L represents the longest diameter and W represents the shortest diameter. Tumor weights were simultaneously measured and recorded at the endpoint. The animal procedures were reviewed and authorized by the Animal Ethics Committee of Chengdu Medical College under approval number Chengdu Medical College Ethics Committee [2026] No. 026.

### 2.14. Hematoxylin and Eosin Staining

Fixed tumor specimens were paraffin-embedded and sectioned for hematoxylin and eosin staining. Paraffin was removed by two successive 15-min incubations in xylene. Tissue sections were then hydrated sequentially in 100%, 95%, 85%, and 75% ethanol, with each step lasting 5 min, and subsequently washed in distilled water for 5 min. Nuclear staining was carried out with hematoxylin for 5 min, after which residual stain was removed by washing with distilled water. The slides were differentiated for 30 s and placed under running tap water for 5 min. Cytoplasmic counterstaining was then performed with eosin for 1 min, followed by removal of excess staining solution. Dehydration was achieved by rapidly passing the slides through 75%, 85%, 95%, and absolute ethanol I for 2–3 s per step, followed by a 1-min incubation in absolute ethanol II. Finally, the specimens underwent two 1-min xylene-clearing steps, were coverslipped with neutral mounting medium, and were examined under a light microscope.

### 2.15. Immunohistochemistry

Target tissues were collected from mice in each group, fixed in 4% paraformaldehyde (Servicebio, Wuhan, China; G1101-500ML), subjected to gradient dehydration, and embedded in paraffin, followed by the preparation of 4-μm-thick serial sections. After deparaffinization and rehydration, the sections were subjected to antigen retrieval and blocking according to the experimental protocol. The blocking solution was removed, and the sections were incubated separately with primary antibodies against Ki67 (KeyGEN BioTECH, Nanjing, China; KGA9402-50) and Cleaved-Caspase3 (CST, Danvers, MA, USA; 9661) overnight at 4 °C. On the following day, the sections were incubated with the corresponding secondary antibody for 1 h at room temperature. DAB staining solution (Zhongshan Golden Bridge Biotechnology, Beijing, China; ZLI-9017) was subsequently applied for color development, and the cell nuclei were counterstained with hematoxylin. After dehydration and clearing, the sections were mounted with neutral resin. Images were captured under a light microscope, and multiple randomly selected fields were examined. The levels of positive protein expression were subsequently analyzed.

### 2.16. Statistical Analysis

Statistical calculations and graphical preparation were carried out in GraphPad Prism 10.1.2. Quantitative data were reported as mean  ±  SD and were obtained from no fewer than three independent biological experiments. For comparisons between two groups, a two-sided Student’s *t*-test was used when the data conformed to a normal distribution; otherwise, significance was assessed with the Mann–Whitney U test. Differences among three or more groups were examined by one-way analysis of variance followed by Tukey’s multiple-comparison test. Relationships between gene expression variables were evaluated using Spearman’s rank correlation coefficient. A *p* value below 0.05 was regarded as evidence of statistical significance.

## 3. Results

### 3.1. ARMCX3 Is Significantly Upregulated in Breast Cancer and Is Associated with Poorer Overall Survival

**Figure 1:**
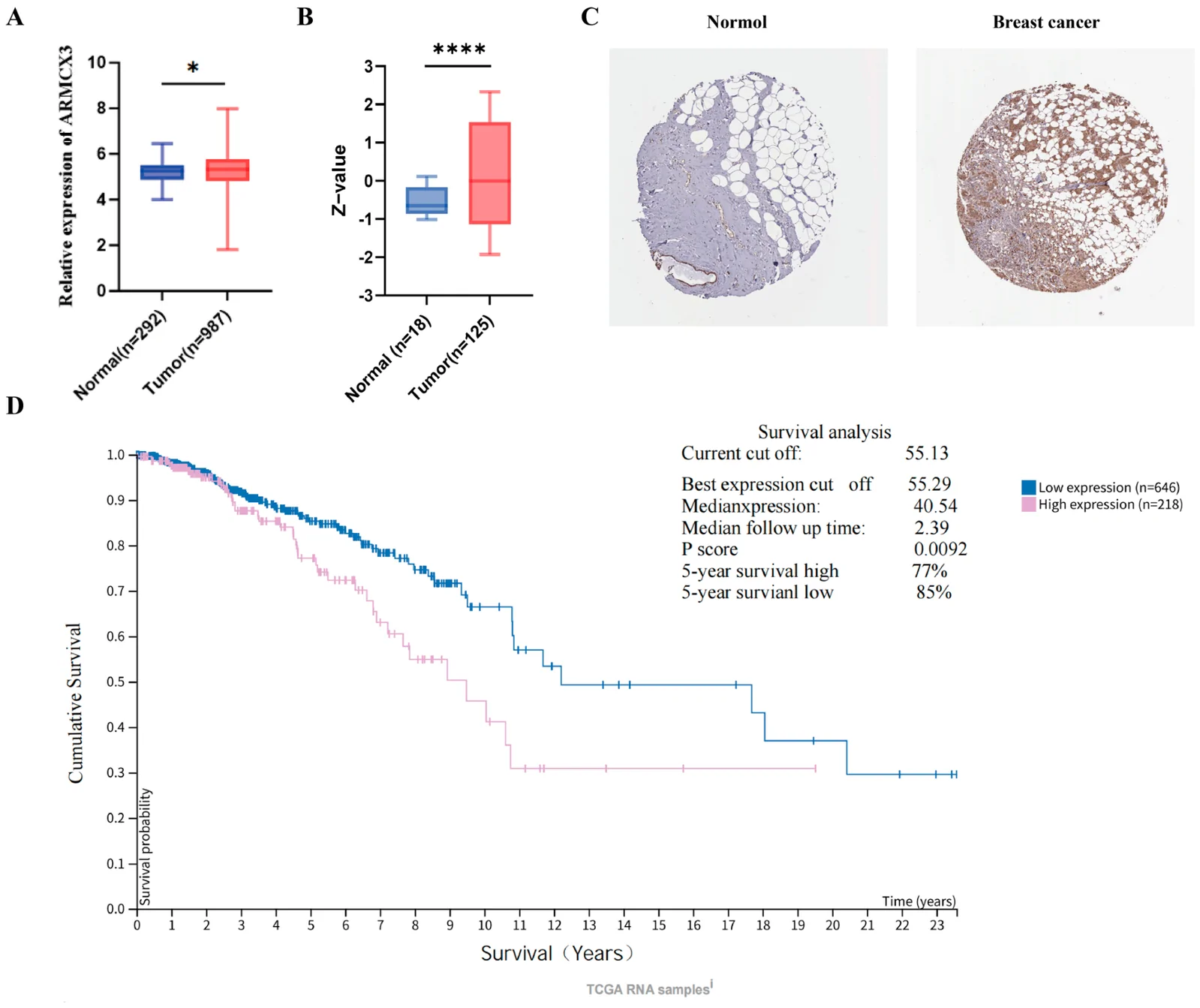
*ARMCX3* is upregulated in breast cancer and is associated with poor overall survival. (**A**) Comparison of *ARMCX3* mRNA expression between normal breast tissues from the GTEx database and breast cancer tissues from the TCGA database. (**B**) Comparison of ARMCX3 protein expression between normal breast tissues and breast cancer tissues based on CPTAC proteomic data analyzed using the UALCAN platform. (**C**) Representative immunohistochemical staining images of ARMCX3 in normal breast tissues and breast cancer tissues obtained from the Human Protein Atlas (HPA) database. (**D**) Kaplan–Meier overall survival analysis of breast cancer patients stratified according to ARMCX3 expression. * *p* < 0.05; **** *p* < 0.0001.

Publicly accessible cancer datasets were used to evaluate the expression pattern and survival association of *ARMCX3* in breast malignancy. Integrated analysis of TCGA and GTEx data showed a marked elevation of *ARMCX3* transcripts in tumor samples relative to nonmalignant breast tissues (*p* = 0.0120, Figure 1A). At the protein level, interrogation of CPTAC data through the UALCAN portal revealed a similar increase in breast carcinoma specimens (*p* < 0.0001, Figure 1B). This expression pattern was further supported by representative immunohistochemical sections obtained from the Human Protein Atlas, which showed higher ARMCX3 protein expression in breast cancer tissues than in normal mammary tissues (Figure 1C). Survival analysis using Kaplan–Meier curves showed that elevated ARMCX3 expression was associated with poorer overall survival compared with lower expression levels (*p* = 0.0092, Figure 1D).

### 3.2. Knockdown of ARMCX3 Suppresses Breast Cancer Cells Proliferation, Migration, and Invasion and Enhances Cisplatin Sensitivity In Vitro

To investigate the role of *ARMCX3* in breast cancer, stable *ARMCX3* knockdown MCF-7 and MDA-MB-231 cells were established using lentivirus-mediated shRNA. Western blotting showed markedly reduced ARMCX3 protein levels in shARMCX3 cells compared with shControl cells (Figure S1A), while RNA-seq analysis revealed reductions of approximately 60% and 70% in *ARMCX3* mRNA levels (FPKM) in MCF-7 and MDA-MB-231 cells, respectively (Figure S1B). To assess the on-target specificity of *ARMCX3* depletion, an shRNA-resistant *ARMCX3* expression construct (*ARMCX3*^Res^) was introduced into *ARMCX3*-depleted cells. Expression of Flag-tagged *ARMCX3*^Res^ was confirmed by Western blotting (Figure S1C). Further Western blot analysis showed that *ARMCX3* re-expression restored total ARMCX3 protein levels to levels comparable to those in shControl cells (Figure S1C), establishing the knockdown and re-expression models for subsequent analyses.

**Figure 2:**
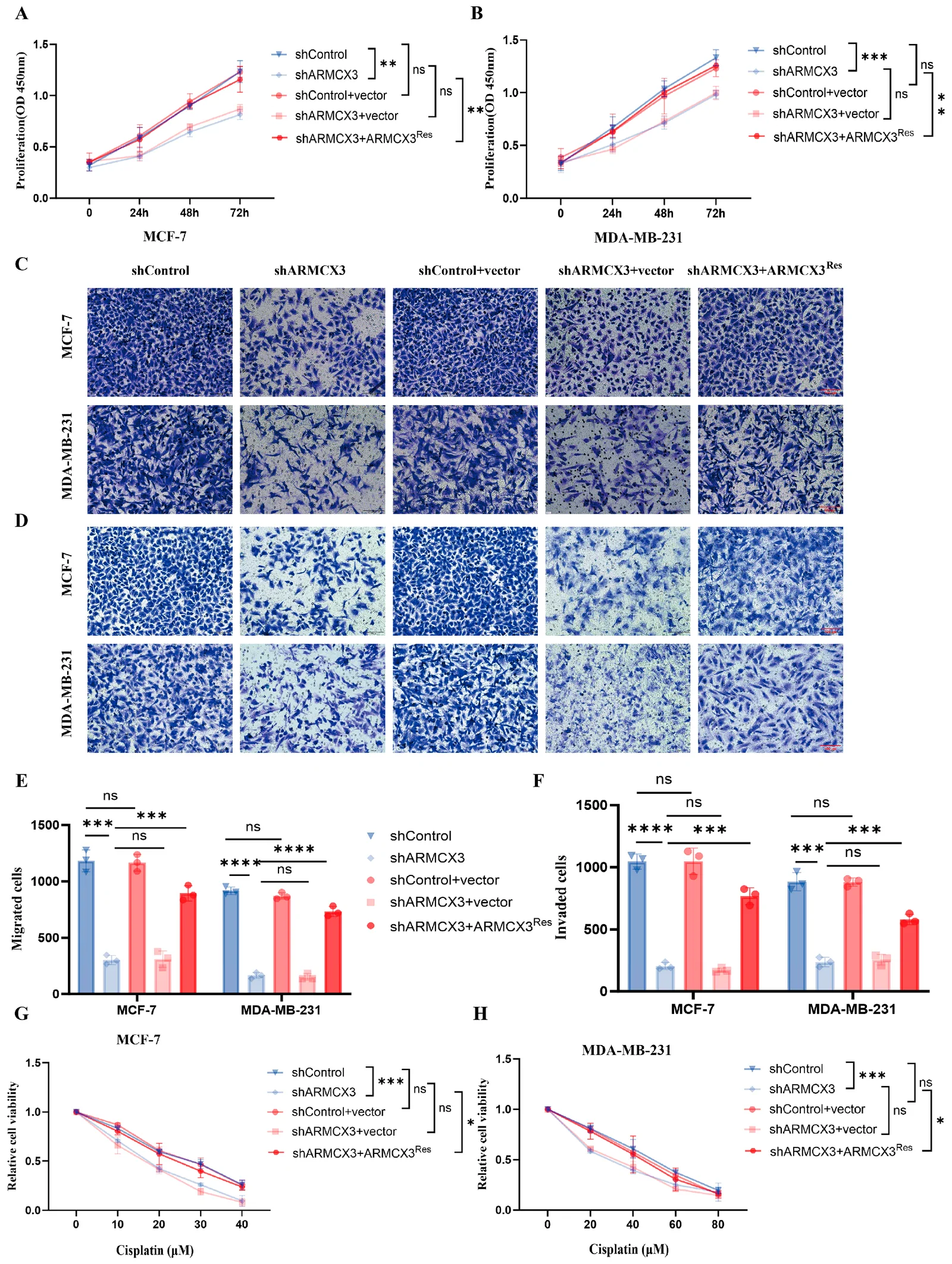
Knockdown of *ARMCX3* inhibits proliferation, migration, and invasion and increases cisplatin sensitivity in breast cancer cells. (**A**,**B**) CCK-8 proliferation assays for shControl, shARMCX3, shControl + vector, shARMCX3 + vector and shARMCX3 + *ARMCX3*^Res^ cells in MCF-7 and MDA-MB-231. (**C**,**D**) Representative images of Transwell migration and invasion assays. Scale bar: 100 μm. (**E**,**F**) Quantitative statistics of migrated and invaded cell numbers. (**G**,**H**) Relative cell viability of the indicated groups exposed to gradient concentrations of cisplatin for 24 h. Data are presented as mean ± SD; ns, not significant; * *p* < 0.05, ** *p* < 0.01, *** *p* < 0.001, **** *p* < 0.0001. Exact *p* values for the indicated statistical comparisons are provided in Table S2.

*ARMCX3* depletion significantly reduced cell proliferation in both MCF-7 and MDA-MB-231 cells, whereas *ARMCX3*^Res^ re-expression partially restored the proliferative capacity of *ARMCX3*-depleted cells (Figure 2A,B). Similarly, *ARMCX3* knockdown markedly reduced cell migration and invasion, as assessed by transwell assays, while *ARMCX3*^Res^ re-expression restored both phenotypes (Figure 2C–F). We next examined the effect of *ARMCX3* depletion on cisplatin sensitivity. Following treatment with increasing concentrations of cisplatin, *ARMCX3* knockdown MCF-7 and MDA-MB-231 cells showed a greater reduction in cell viability than their corresponding controls, indicating increased cisplatin sensitivity in vitro; however, *ARMCX3*^Res^ re-expression restored cell viability following cisplatin treatment (Figure 2G,H).

Collectively, *ARMCX3* depletion suppresses breast cancer cell proliferation, migration, and invasion while enhancing cisplatin sensitivity in vitro.

### 3.3. ARMCX3 Knockdown Is Associated with Alterations in p53-Associated Signaling as Revealed by Transcriptomic Profiling

**Figure 3:**
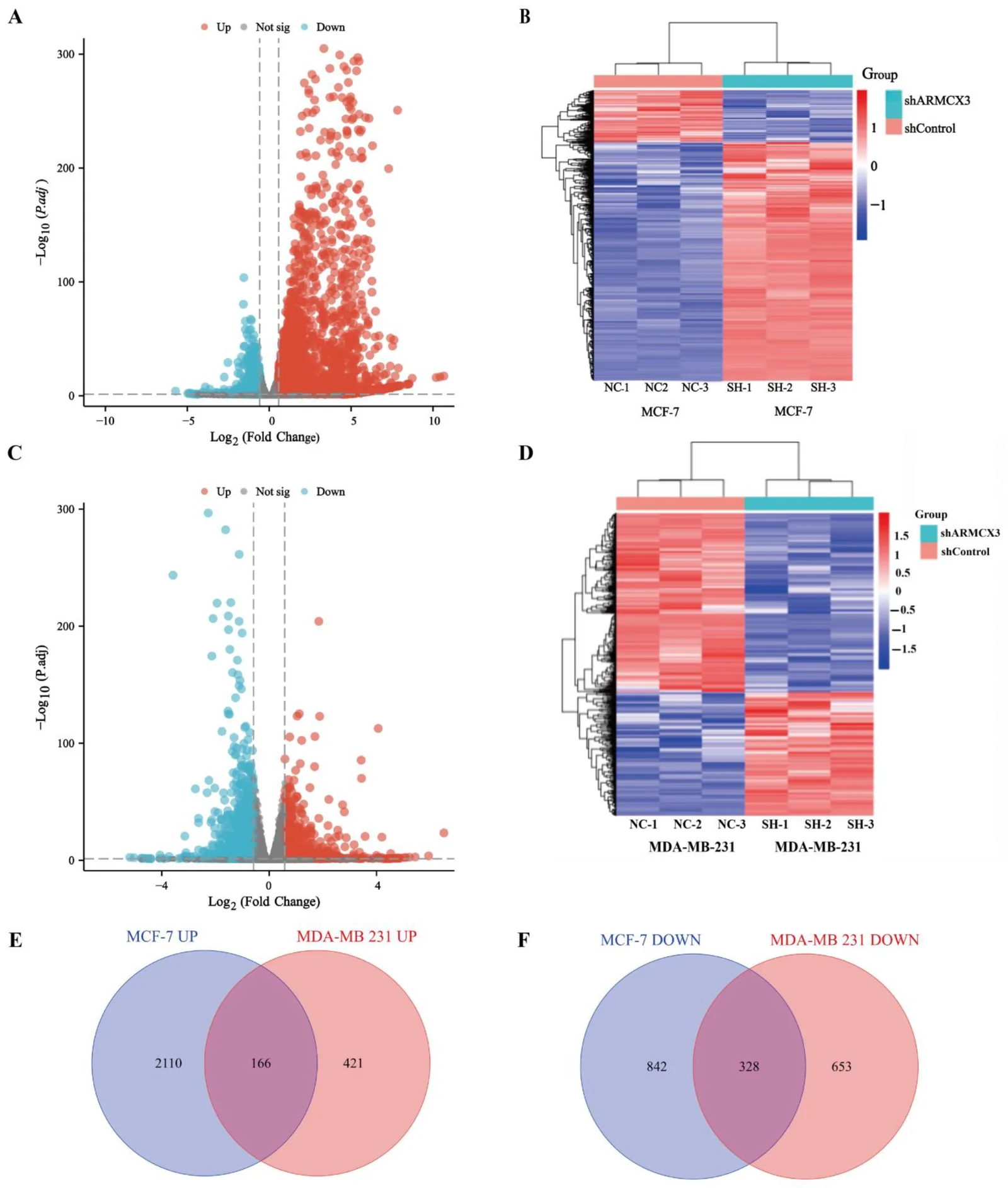
Transcriptomic profiling of breast cancer cells following *ARMCX3* knockdown. (**A**,**C**) Volcano plots displaying differentially expressed genes (DEGs) in MCF-7 (A) and MDA-MB-231 (**C**) cells upon *ARMCX3* knockdown. (**B**,**D**) Hierarchical clustering heatmaps showing the expression profiles of the DEGs in MCF-7 (**B**) and MDA-MB-231 (**D**) cells. (**E**,**F**) Venn diagrams showing overlapping upregulated (**E**) and downregulated (**F**) DEGs between two cell lines.

To investigate the molecular changes associated with *ARMCX3* depletion, RNA sequencing was performed in MCF-7 and MDA-MB-231 cells expressing shControl or shARMCX3. In MCF-7 cells, 3446 genes showed significant expression alterations, of which 2276 displayed increased expression and 1170 displayed decreased expression (Figure 3A,B,E,F). In MDA-MB-231 cells, 1568 genes met the differential-expression criteria, with 587 showing positive expression changes and 981 showing negative expression changes (Figure 3C–F).

**Figure 4:**
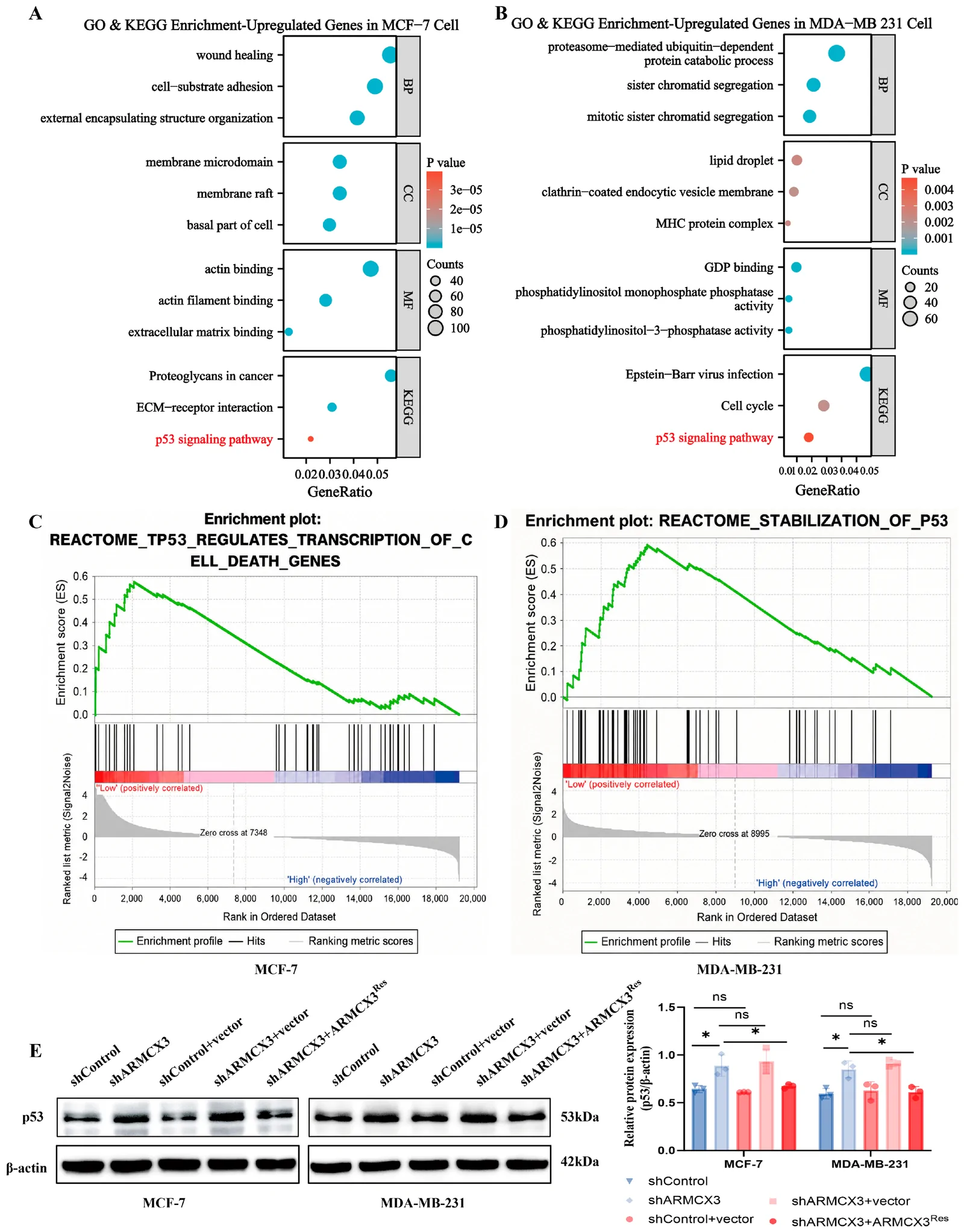
Enrichment of p53-associated signaling following *ARMCX3* knockdown and validation of p53 protein abundance. (**A**,**B**) GO and KEGG enrichment analyses of upregulated genes in MCF-7 (**A**) and MDA-MB-231 (**B**) cells following *ARMCX3* knockdown. (**C**,**D**) GSEA plots showing the enrichment of p53-related gene sets in MCF-7 (**C**) and MDA-MB-231 (**D**) cells upon *ARMCX3* knockdown. The pink-to-blue gradient represents the ranking metric, from genes positively associated with the low-*ARMCX3* phenotype to genes negatively associated with it; black vertical lines indicate the positions of genes in each gene set within the ranked list. (**E**) Western blot analysis and quantification of p53 protein levels in shControl, shARMCX3, shControl + vector, shARMCX3 + vector and shARMCX3 + *ARMCX3*^Res^ MCF-7 and MDA-MB-231 cells. For rescue experiments, shARMCX3 cells were transfected with an shRNA-resistant *ARMCX3* construct (*ARMCX3*^Res^), while vector served as the matched empty control. Data are presented as mean ± SD. ns, not significant; * *p* < 0.05. Exact *p* values for the indicated statistical comparisons are provided in Table S3.

Functional annotation of these gene sets was then carried out through Gene Ontology and Kyoto Encyclopedia of Genes and Genomes pathway analyses. The resulting enrichment profiles from both cell models highlighted the p53 signaling cascade as one of the most prominently altered pathways following *ARMCX3* depletion (Figure 4A,B). Gene set enrichment analysis further showed enrichment of p53-related gene sets following *ARMCX3* depletion in both MCF-7 and MDA-MB-231 cells (Figure 4C,D). Consistent with these enrichment results, further analysis of the RNA-sequencing data showed increased expression of several established p53 target genes, including *CDKN1A*, *FAS*, *TP53I3*, *RPRM*, *PHLDA3*, *AIFM2*, and *DDB2*, following *ARMCX3* depletion in both cell models (Figure S2A,B). Notably, RNA-sequencing-derived *CDKN1A* expression was significantly increased in both MCF-7 and MDA-MB-231 cells following *ARMCX3* depletion (Figure S2C,D).

Consistent with these transcriptomic changes, immunoblot analysis showed increased p53 protein abundance following *ARMCX3* depletion (Figure 4E). Importantly, re-expression of the shRNA-resistant *ARMCX3* construct reversed the increase in p53 protein levels induced by *ARMCX3* depletion (Figure 4E). Together, these findings indicate that *ARMCX3* depletion is associated with alterations in p53-related signaling in both breast cancer cell models.

### 3.4. Pharmacological Treatment with PFT-α Attenuates Phenotypic and p53-Associated Changes Following ARMCX3 Knockdown in Breast Cancer Cells

**Figure 5:**
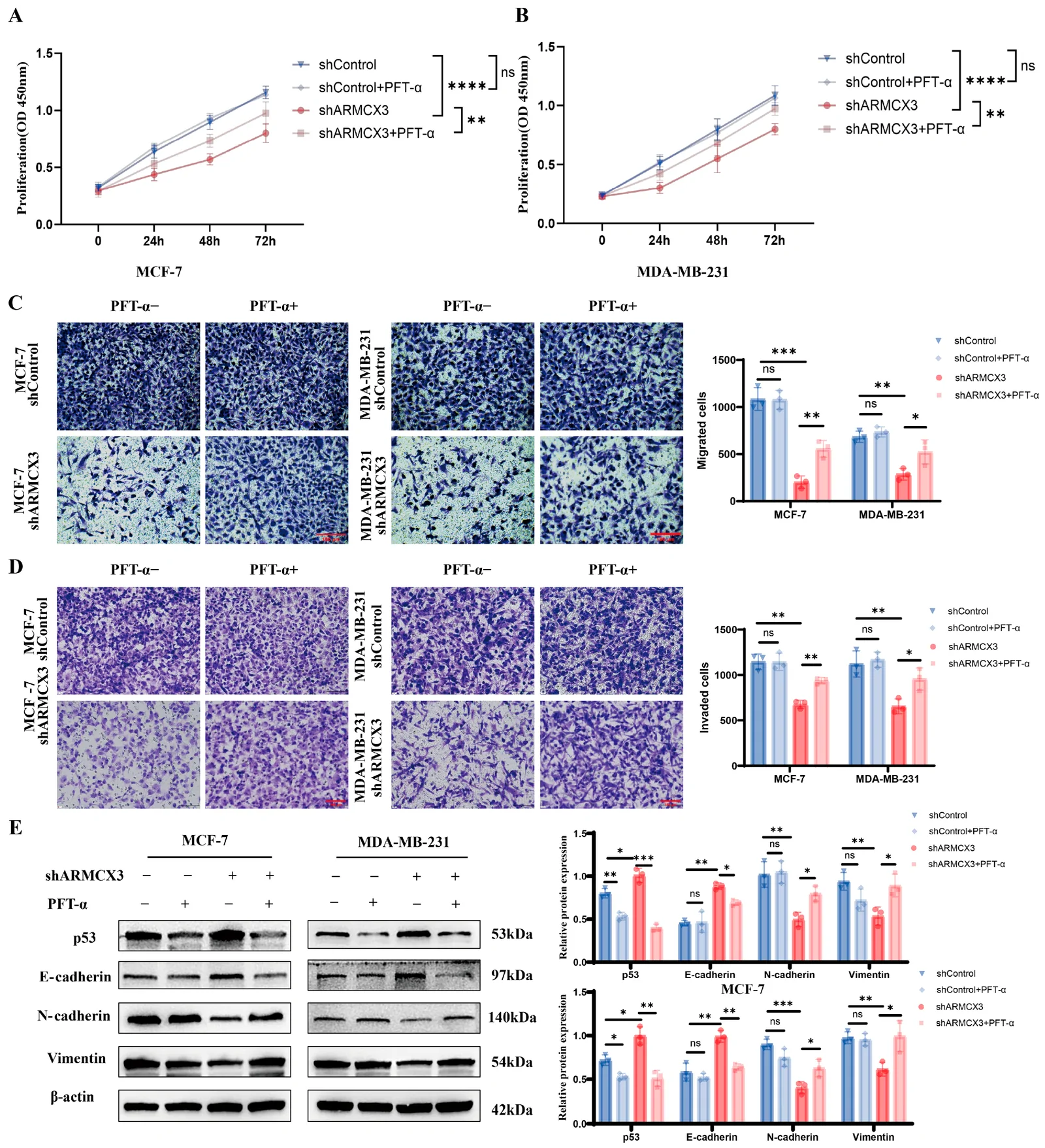
PFT-α treatment attenuates the effects of *ARMCX3* knockdown on breast cancer cell proliferation, migration, invasion, and EMT-associated protein expression. (**A**,**B**) CCK-8 assays showing the proliferation of shControl and shARMCX3 MCF-7 (**A**) and MDA-MB-231 (**B**) cells treated with or without the p53 inhibitor PFT-α for 0, 24, 48, and 72 h. (**C**,**D**) Representative Transwell images and quantitative analysis of migrated (**C**) and invaded (**D**) MCF-7 and MDA-MB-231 cells following treatment with 20 μM PFT-α (MCF-7) or 30 μM PFT-α (MDA-MB-231) for 48 h. Scale bar: 100 μm. (**E**) Western blot analysis and corresponding quantitative analysis of p53, E-cadherin, N-cadherin, and vimentin protein expression in shControl and shARMCX3 MCF-7 and MDA-MB-231 cells treated with 20 μM PFT-α (MCF-7) or 30 μM PFT-α (MDA-MB-231) for 48 h. Data are presented as mean ± SD. ns, not significant; * *p* < 0.05, ** *p* < 0.01, *** *p* < 0.001, **** *p* < 0.0001. Exact *p* values for the indicated statistical comparisons are provided in Table S4.

We next investigated whether p53-associated signaling contributes to the phenotypic changes following *ARMCX3* knockdown. To this end, cells were treated with the PFT-α, a commonly used pharmacological inhibitor of p53 transcriptional activity. CCK-8 assays showed that *ARMCX3* knockdown significantly inhibited the proliferation of both MCF-7 and MDA-MB-231 cells, whereas PFT-α treatment attenuated this reduction in cell proliferation (Figure 5A,B). Similarly, Transwell assays demonstrated that *ARMCX3* depletion markedly reduced the migratory and invasive capacities of both breast cancer cell lines, and these effects were attenuated by PFT-α treatment (Figure 5C,D).

The expression of EMT-related proteins was also examined by Western blotting (Figure 5E). Compared with the control group, *ARMCX3* knockdown increased E-cadherin expression while reducing N-cadherin and vimentin expression, indicating inhibition of EMT. Following PFT-α treatment, these changes were largely reversed, with decreased E-cadherin expression and restored expression of the mesenchymal markers N-cadherin and vimentin.

**Figure 6:**
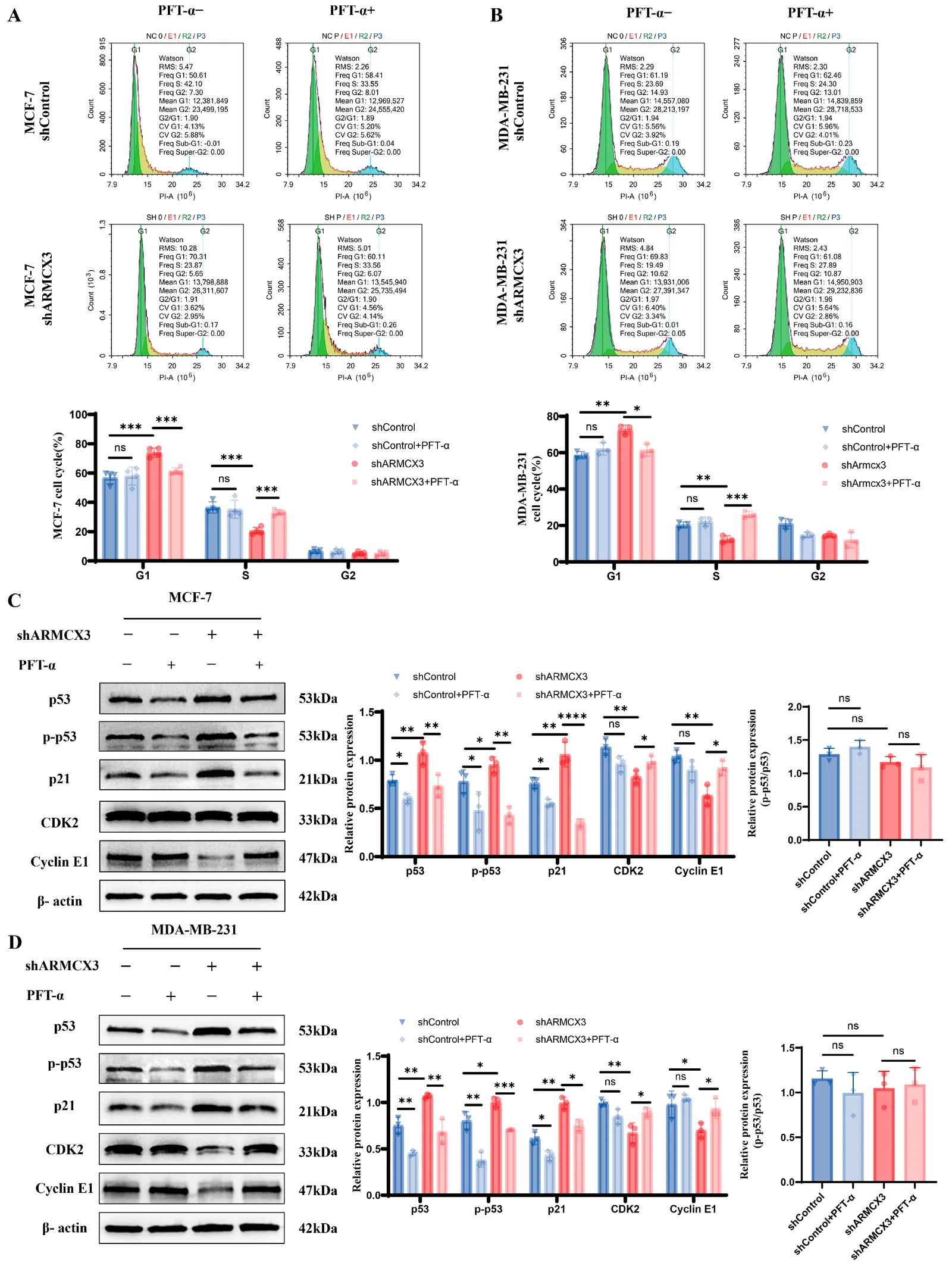
PFT-α-mediated p53 inhibition partially reverses *ARMCX3* knockdown-induced G1-phase cell-cycle arrest and the associated changes in cell-cycle regulatory proteins. (**A**,**B**) Representative flow cytometric histograms and quantitative analysis of cell-cycle distribution in shControl and shARMCX3 MCF-7 (**A**) and MDA-MB-231 (**B**) cells treated with 20 μM PFT-α (MCF-7) or 30 μM PFT-α (MDA-MB-231) for 48 h. In the cell-cycle histograms, the green, yellow, and light blue areas represent the G1, S, and G2/M phases, respectively. (**C**,**D**) Western blot analysis and corresponding quantitative analysis of p53, phosphorylated p53 (p-p53), p21, CDK2, and Cyclin E1 protein expression in shControl and shARMCX3 MCF-7 (**C**) and MDA-MB-231 (**D**) cells following treatment with 20 μM PFT-α (MCF-7) or 30 μM PFT-α (MDA-MB-231) for 48 h. The p-p53/p53 ratio was additionally quantified to evaluate the relative phosphorylation level of p53. Data are presented as mean ± SD. ns, not significant; * *p* < 0.05, ** *p* < 0.01, *** *p* < 0.001, **** *p* < 0.0001. Exact *p* values for the indicated statistical comparisons are provided in Table S5.

Because inhibition of cell proliferation is commonly associated with cell-cycle arrest, we next examined cell-cycle distribution by flow cytometry [23]. *ARMCX3* knockdown significantly increased the proportion of cells in the G1 phase while reducing the S-phase population in both breast cancer cell lines (Figure 6A,B), while PFT-α treatment attenuated these alterations, supporting the involvement of p53-associated signaling in the G1-phase arrest observed following *ARMCX3* depletion. To further investigate the molecular basis of this phenotype, we then examined key regulators of the G1/S transition. Western blot analysis showed increased total and phosphorylated p53 abundance following *ARMCX3* depletion, and consistent with the increased *CDKN1A* transcript abundance observed in the RNA-sequencing analysis (Figure S2C,D), p21 protein abundance was also increased following *ARMCX3* depletion, whereas the total protein levels of CDK2 and Cyclin E1 were decreased (Figure 6C,D). The p-p53/p53 ratio was not significantly altered, indicating that the increase in phosphorylated p53 was proportional to the accumulation of total p53. Notably, PFT-α treatment attenuated the *ARMCX3* knockdown-associated increase in p53 and p21 protein expression and restored CDK2 and Cyclin E1 expression.

Together, these findings indicate that *ARMCX3* depletion is associated with G1-phase arrest accompanied by p53 accumulation, p21 upregulation, and reduced abundance of CDK2 and Cyclin E1. Interestingly, the attenuation of these phenotypic and molecular changes by PFT-α provides pharmacological evidence supporting the involvement of p53-associated signaling in the effects of *ARMCX3* knockdown on breast cancer cell proliferation, migration, invasion, EMT-related changes, and G1/S cell-cycle progression.

### 3.5. ARMCX3 Knockdown Enhances Cisplatin-Induced Apoptosis and DNA Damage In Vitro in Association with p53 Signaling

**Figure 7:**
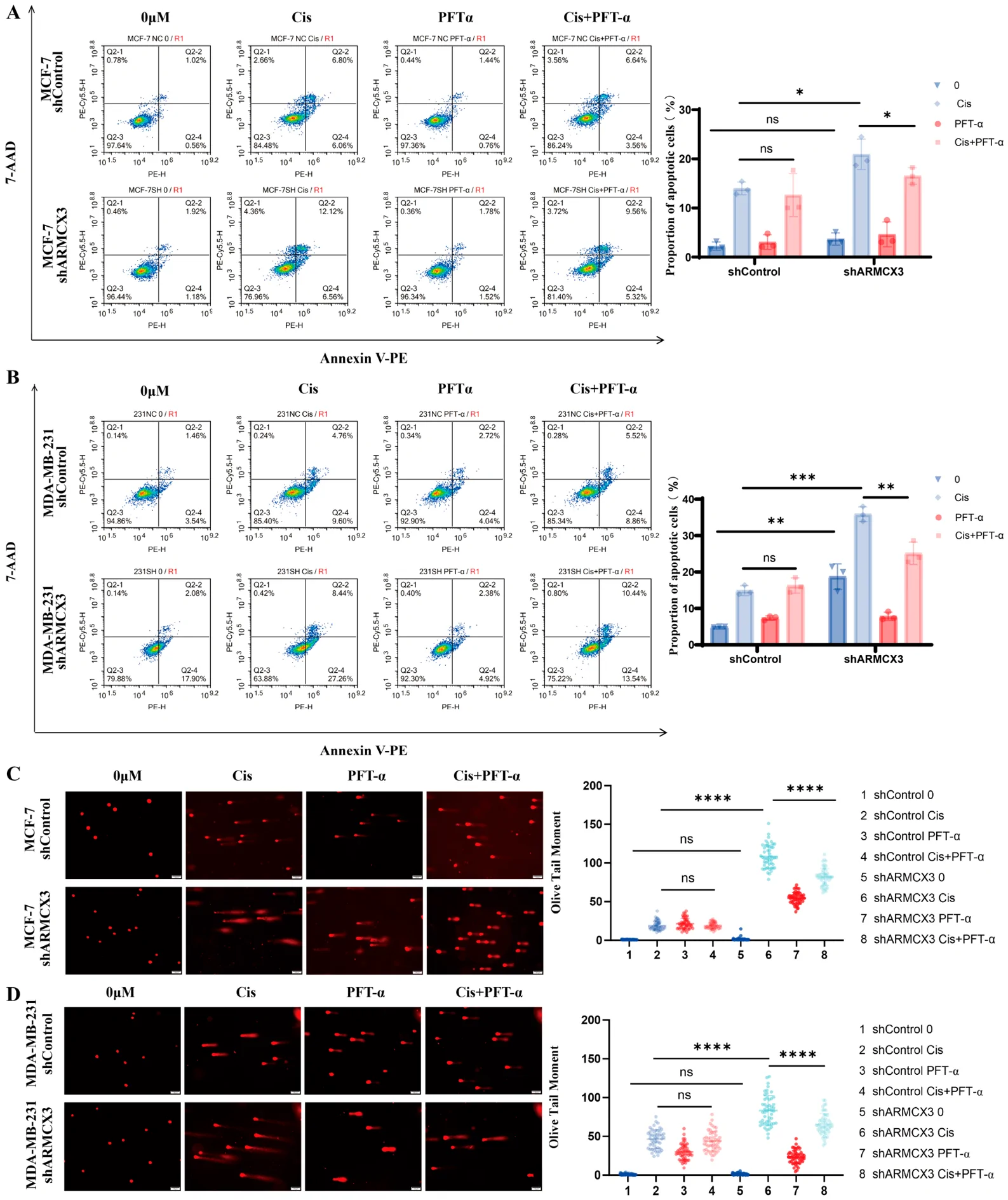
PFT-α attenuates the enhanced cisplatin-induced apoptosis and DNA damage associated with *ARMCX3* knockdown in breast cancer cells. (**A**,**B**) Flow cytometric analysis and quantitative analysis of apoptosis in shControl and shARMCX3 MCF-7 (**A**) and MDA-MB-231 (**B**) cells following treatment with cisplatin and/or PFT-α. MCF-7 cells were treated with cisplatin (10 μM) and/or PFT-α (20 μM), whereas MDA-MB-231 cells were treated with cisplatin (20 μM) and/or PFT-α (30 μM) for 24 h. In the flow cytometry dot plots, the different colors indicate cell populations with varying fluorescence intensities, with red and yellow representing regions of higher cell density and blue representing regions of lower cell density. (**C**,**D**) Representative comet assay images and quantitative analysis of olive tail moment (OTM) in shControl and shARMCX3 MCF-7 (**C**) and MDA-MB-231 (**D**) cells following treatment with cisplatin and/or PFT-α. MCF-7 cells were exposed to cisplatin (10 μM) and/or PFT-α (20 μM), whereas MDA-MB-231 cells were exposed to cisplatin (20 μM) and/or PFT-α (30 μM) for 24 h. Scale bar: 50 μm. Data are presented as mean ± SD. ns, not significant; * *p* < 0.05, ** *p* < 0.01, *** *p* < 0.001, **** *p* < 0.0001. Exact *p* values for the indicated statistical comparisons are provided in Table S6.

**Figure 8:**
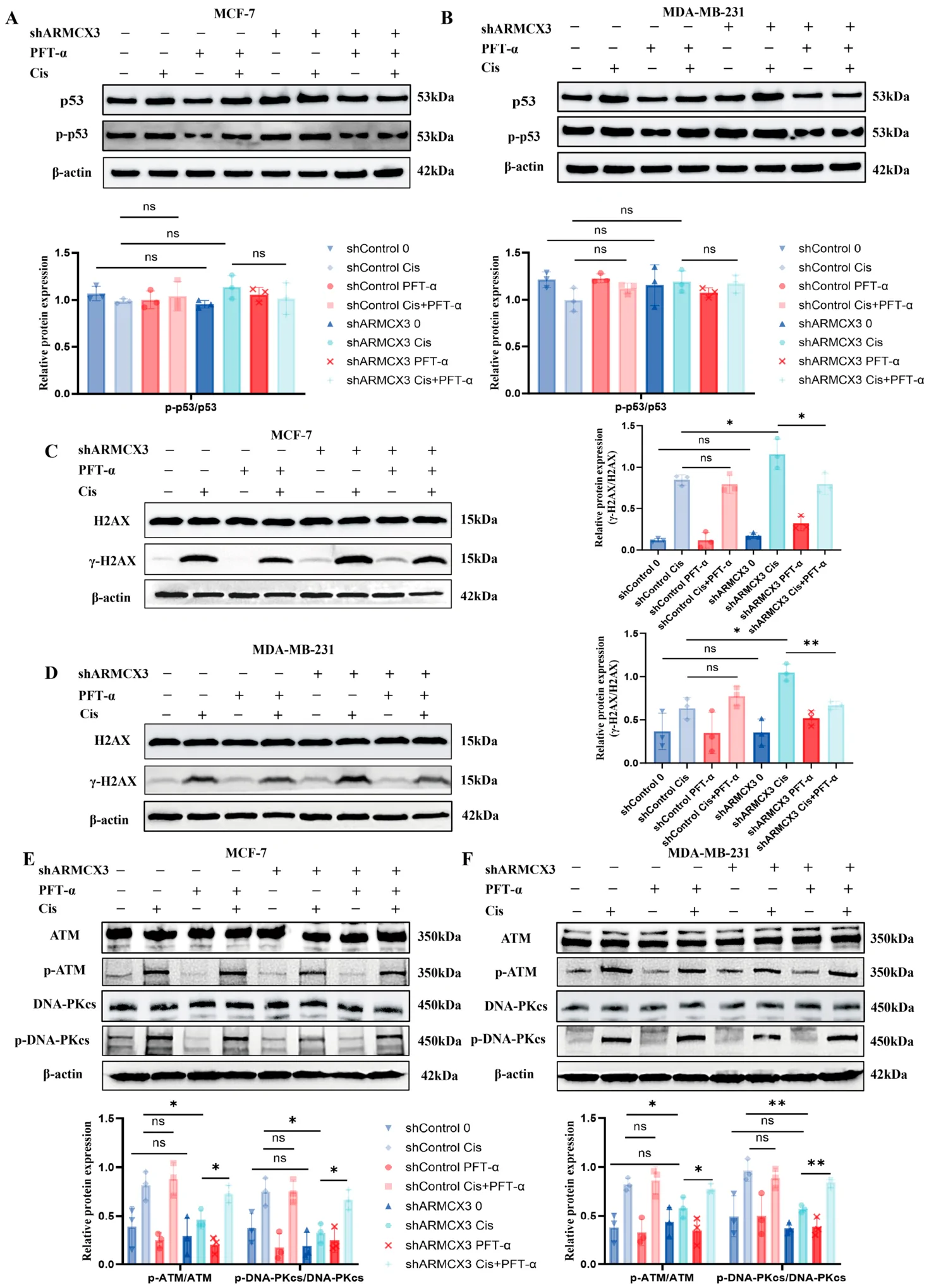
Changes in p53 and DNA damage response-related proteins following cisplatin and PFT-α treatment in *ARMCX3* knockdown breast cancer cells. (**A**,**B**) Western blot analysis of p53 and p-p53 in shControl and shARMCX3 MCF-7 (**A**) and MDA-MB-231 (**B**) cells under the indicated treatment conditions, with quantification of the p-p53/p53 ratios. (**C**,**D**) Western blot analysis of total H2AX and γ-H2AX in MCF-7 (**C**) and MDA-MB-231 (**D**) cells under the indicated treatment conditions, with quantification of the γ-H2AX/H2AX ratio. (**E**,**F**) Western blot analysis of total and phosphorylated ATM and DNA-PKcs, together with the p-ATM/ATM and p-DNA-PKcs/DNA-PKcs ratios, in MCF-7 (**E**) and MDA-MB-231 (**F**) cells under the indicated treatment conditions, with quantification of the p-ATM/ATM and p-DNA-PKcs/DNA-PKcs ratios. For all panels, MCF-7 cells were treated with cisplatin (10 μM) and/or PFT-α (20 μM), whereas MDA-MB-231 cells were treated with cisplatin (20 μM) and/or PFT-α (30 μM) for 24 h. Data are presented as mean ± SD. ns, not significant; * *p* < 0.05, ** *p* < 0.01. Exact *p* values for the indicated statistical comparisons are provided in Table S7. Densitometric quantification of the individual proteins normalized to β-actin is provided in Figure S3.

Cisplatin is a commonly used chemotherapeutic agent in breast cancer treatment [5,24]. We next investigated whether p53-associated signaling is involved in the enhanced cisplatin response following *ARMCX3* depletion. Cells were co-treated with PFT-α and cisplatin to assess the chemosensitizing effect of *ARMCX3* depletion. Flow cytometric analysis showed that cisplatin treatment significantly increased apoptosis in both MCF-7 and MDA-MB-231 cells (Figure 7A,B). *ARMCX3* knockdown further enhanced cisplatin-induced apoptosis, whereas PFT-α significantly attenuated this effect in *ARMCX3* knockdown cells but did not significantly affect cisplatin-induced apoptosis in shControl cells. Because cisplatin induces DNA lesions, particularly DNA crosslinks that can subsequently lead to DNA strand breaks [25], we next evaluated DNA damage under the same treatment conditions. Comet assays revealed increased DNA fragmentation in *ARMCX3* knockdown cells following cisplatin exposure, as evidenced by increased comet tailing and Olive tail moment. PFT-α significantly attenuated these changes in *ARMCX3* knockdown cells but had no significant effect in shControl cells (Figure 7C,D). Western blotting showed that cisplatin treatment increased protein levels of p53 and p-p53 in *ARMCX3* knockdown cells, while PFT-α attenuated these increases (Figure 8A,B; Figure S3A,B). Consistent with the comet assay, *ARMCX3* knockdown increased γ-H2AX accumulation following cisplatin treatment in both MCF-7 and MDA-MB-231 cells, whereas PFT-α reduced γ-H2AX accumulation in *ARMCX3* knockdown cells (Figure 8C,D; Figure S3C,D).

To further characterize DNA damage response signaling associated with the increased DNA damage accumulation, we examined ATM and DNA-PKcs, two key kinases involved in DNA double-strand break responses and repair-associated signaling. Following cisplatin treatment, *ARMCX3* knockdown reduced p-ATM and p-DNA-PKcs levels and the corresponding p-ATM/ATM and p-DNA-PKcs/DNA-PKcs ratios, without markedly affecting total ATM or DNA-PKcs levels (Figure 8E,F; Figure S3E,F). PFT-α partially restored ATM and DNA-PKcs phosphorylation and their corresponding ratios in *ARMCX3* knockdown cells.

Collectively, these findings indicate that *ARMCX3* depletion is associated with enhanced cisplatin-induced DNA damage and apoptosis in vitro, and the attenuation of these effects by PFT-α further supports the involvement of p53-associated signaling in this response.

### 3.6. ARMCX3 Knockdown Suppresses Breast Tumor Growth In Vivo and This Effect Is Attenuated by PFT-α

**Figure 9:**
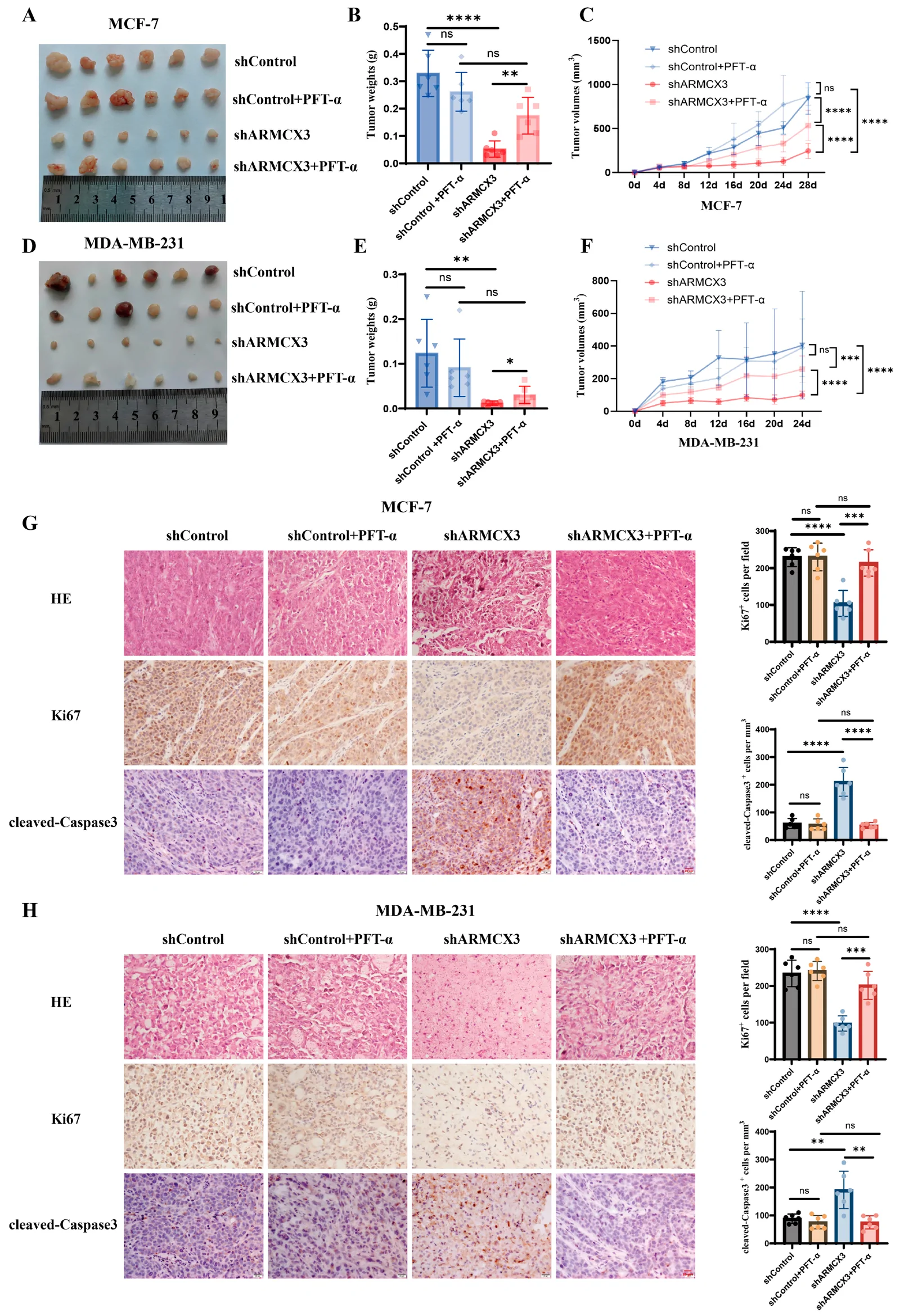
*ARMCX3* knockdown suppresses breast cancer xenograft growth, and these effects are attenuated by PFT-α. (**A**,**D**) Representative images of xenograft tumors formed by MCF-7 (**A**) and MDA-MB-231 (**D**) cells in nude mice under different treatments. (**B**,**E**) Tumor weights of MCF-7 (**B**) and MDA-MB-231 (**E**) xenografts in each group. (**C**,**F**) Tumor volume growth curves of MCF-7 (**C**) and MDA-MB-231 (**F**) xenografts over time. *n* = 6 mice per group. (**G**,**H**) Representative images of HE staining, Ki67 and cleaved-Caspase3 immunohistochemical staining in MCF-7 (**G**) and MDA-MB-231 (**H**) xenograft tissues, with corresponding quantitative analysis. Scale bar: 50 μm. Data are presented as mean ± SD. ns, not significant; * *p* < 0.05, ** *p* < 0.01, *** *p* < 0.001, **** *p* < 0.0001. Exact *p* values for the indicated statistical comparisons are provided in Table S8.

To evaluate the effect of *ARMCX3* depletion on breast tumor growth in vivo and further examine the pharmacological effects of PFT-α in this context, breast cancer cells with stable *ARMCX3* silencing were implanted subcutaneously, and tumor development was monitored. Relative to the shControl group, xenografts formed by *ARMCX3*-deficient cells showed a pronounced reduction in size and final mass; however, administration of PFT-α substantially weakened this growth suppression (Figure 9A–F).

We next performed histological and immunohistochemical analyses of xenograft tumors. Hematoxylin and eosin (H&E) staining revealed disrupted tumor architecture and reduced cellular density in tumors from the *ARMCX3* knockdown group, whereas these histological alterations were partially alleviated following PFT-α treatment (Figure 9G,H). Consistent with the reduced tumor growth, immunohistochemical staining demonstrated that *ARMCX3* knockdown markedly decreased the number of Ki67-positive cells and increased cleaved-Caspase3 immunoreactivity in xenograft tissues. PFT-α treatment attenuated these changes, increasing Ki67-positive staining and reducing cleaved-Caspase3 immunoreactivity toward the levels observed in the control group. In summary, PFT-α treatment attenuated the suppression of xenograft growth and the associated changes in Ki67-positive staining and cleaved-Caspase3 immunoreactivity.

## 4. Discussion

Despite advances in systemic treatment, breast malignancy continues to account for substantial cancer mortality in women, while acquired resistance frequently compromises the clinical benefit of platinum-containing regimens [2,5]. In the present study, we demonstrated that *ARMCX3* depletion suppressed malignant phenotypes and enhanced cisplatin sensitivity in breast cancer cells, with these effects being reversed by *ARMCX3* re-expression. Transcriptomic profiling combined with molecular assays identified p53-associated signaling as a prominent molecular response following *ARMCX3* depletion in breast cancer cells. Treatment with PFT-α attenuated several phenotypic and molecular changes associated with *ARMCX3* knockdown, providing pharmacological evidence consistent with the involvement of p53-associated signaling in these responses. These findings support that p53-associated signaling contributes to the biological effects triggered by *ARMCX3* silencing.

p53 functions as a key integrator of cellular stress signals and governs a broad range of biological processes, including cell-cycle control, programmed cell death, senescence, and the DNA damage response [26,27]. Earlier studies have established that p53 activation can restrain tumor development by promoting p21-dependent arrest at the G1/S checkpoint and by limiting metastatic traits through regulation of epithelial–mesenchymal transition-related programs [28,29]. At the G1/S checkpoint, p21 classically binds to and inhibits Cyclin E–CDK2 complexes, thereby suppressing CDK2 kinase activity and preventing cell-cycle progression [30,31]. Sustained p53–p21 signaling may also maintain RB in a hypophosphorylated state and restrain E2F-dependent transcriptional programs required for entry into S phase [32]. Consistent with this regulatory framework, our transcriptomic analysis showed increased expression of several p53-associated genes, including *CDKN1A*, *FAS*, *TP53I3*, *RPRM*, *PHLDA3*, *AIFM2*, and *DDB2*, following *ARMCX3* depletion in both breast cancer cell models (Figure S2). Notably, increased *CDKN1A* transcript abundance was accompanied by increased p21 protein expression, decreased CDK2 and Cyclin E1 abundance, and G1-phase accumulation. PFT-α attenuated the changes in p21 and other G1/S regulatory proteins as well as the associated cell-cycle phenotype. The concordant changes in *CDKN1A* transcript abundance, p21 protein expression, and G1/S regulators therefore provide downstream molecular evidence consistent with modulation of p53-associated cell-cycle signaling following *ARMCX3* depletion. PFT-α also attenuated the effects of *ARMCX3* depletion on proliferation, migration, invasion, and EMT-associated proteins, further supporting the involvement of p53-associated signaling in these cellular responses.

Beyond its role in cell-cycle regulation, p53 is also a key determinant of cellular responses to DNA-damaging chemotherapy. Following genotoxic stress, p53 coordinates the balance between DNA repair and apoptosis [33]. Moderate DNA damage may initially activate repair pathways, whereas persistent or excessive DNA damage promotes p53-dependent apoptotic signaling to eliminate damaged cells [34]. Given the central role of p53 in cellular responses to DNA damage, we next examined whether this signaling pathway also contributes to the response of *ARMCX3*-depleted cells to cisplatin-induced DNA damage. Previous studies have shown that cisplatin activates p53 signaling and that PFT-α can attenuate cisplatin-induced cellular responses [35,36]. Consistent with these observations, cisplatin treatment increased p53 phosphorylation and induced apoptosis in our breast cancer models, whereas *ARMCX3* depletion further enhanced these responses, as reflected by increased p53 protein abundance and phosphorylation, together with elevated apoptotic rates. *ARMCX3* knockdown was also accompanied by increased γ-H2AX accumulation and greater DNA fragmentation in comet assays, indicating persistent DNA-strand-break-associated signals upon cisplatin exposure, which may stem from elevated lesion induction, compromised damage resolution, or both. In parallel, *ARMCX3* depletion reduced the phosphorylation of ATM and DNA-PKcs without markedly altering their total protein levels, suggesting perturbations in DNA-damage-response signaling. Importantly, PFT-α attenuated the p53-associated molecular changes, DNA damage accumulation, and apoptosis associated with *ARMCX3* depletion. These findings support the involvement of p53-associated signaling in the enhanced cellular response to cisplatin following *ARMCX3* depletion in vitro.

We additionally examined ERK signaling following *ARMCX3* depletion. Changes in ERK1/2 phosphorylation and p53 protein abundance were observed following *ARMCX3* depletion, *ARMCX3* re-expression, and pharmacological MEK inhibition; however, the phenotypic effects of trametinib were not consistent with a clear rescue. Therefore, these findings are presented in the Supplementary Information as exploratory supporting observations (Figures S4–S7).

An interesting observation in this study is that *ARMCX3* knockdown induced p53-associated signaling responses in both MCF-7 cells carrying wild-type *TP53* and MDA-MB-231 cells harboring the *TP53* R280K missense mutation. Wild-type *TP53* primarily functions as a transcription factor that induces cell-cycle arrest and apoptosis, whereas mutant *TP53* frequently acquires oncogenic gain-of-function properties that promote tumor progression and therapeutic resistance [37,38]. Importantly, accumulating evidence indicates considerable functional heterogeneity among p53 mutants, with their transcriptional and biological activities varying according to the specific mutation and cellular context [39,40,41]. In this study, *ARMCX3* depletion increased p53 protein abundance in both cell models. Transcriptomic analysis further showed increased expression of several p53-associated genes, including *CDKN1A*, *FAS*, *TP53I3*, *RPRM*, *PHLDA3*, *AIFM2*, and *DDB2*, with increased *CDKN1A* transcript abundance accompanied by increased p21 protein expression and changes in G1/S cell-cycle regulators. These transcriptional and protein-level changes provide downstream molecular evidence consistent with modulation of p53-associated signaling. In addition, PFT-α attenuated multiple cellular responses associated with *ARMCX3* depletion. The use of PFT-α to functionally investigate p53-associated signaling has also been reported in previous cancer studies using different cellular models, where PFT-α attenuated p53-associated changes in proliferation, cell-cycle progression, apoptosis, and other malignant phenotypes [42,43]. Together, these observations support the involvement of p53-associated signaling in the cellular responses following *ARMCX3* depletion across the two breast cancer models examined. Nevertheless, given the R280K mutation in MDA-MB-231 cells, the increased p53 protein abundance observed in this model should not be interpreted as restoration of canonical wild-type *TP53* function. Rather, our findings indicate that *ARMCX3* depletion is associated with p53-related molecular and phenotypic responses in both wild-type and mutant *TP53* cellular contexts. The specific functional properties of R280K mutant p53 in this regulatory context warrant further investigation and provide a basis for future studies of the relationship between *ARMCX3* and p53 signaling in *TP53*-mutant breast cancer. 

Several limitations of this study should be acknowledged. First, although *ARMCX3* depletion increased the expression of several p53-associated genes and was accompanied by concordant changes in *CDKN1A*/p21 and G1/S regulators, these findings do not establish direct transcriptional regulation by *TP53* R280K or canonical wild-type p53 activity in MDA-MB-231 cells. Moreover, because PFT-α has recognized limitations in specificity, its effects provide pharmacological support rather than definitive evidence for p53 involvement. Genetic *TP53* manipulation will be required to further define its specific contribution. Second, only MCF-7 and MDA-MB-231 cells were examined, and additional models with distinct molecular and *TP53* backgrounds are needed to determine the generalizability of these findings. Third, although the shRNA-resistant *ARMCX3*^Res^ rescue experiments support the on-target specificity of representative effects, a single *ARMCX3*-targeting shRNA was used, and validation with an independent depletion strategy would provide additional support. Fourth, the enhanced cisplatin responsiveness associated with *ARMCX3* depletion was demonstrated in vitro but was not evaluated in the xenograft models. Finally, the expression and survival associations identified using public datasets were not independently validated in a clinically annotated patient cohort. Therefore, further in vivo chemotherapy validation and clinical studies will be required to establish the translational relevance of *ARMCX3* in breast cancer.

In conclusion, this study demonstrates that *ARMCX3* contributes to breast cancer progression and cisplatin responsiveness. *ARMCX3* depletion suppressed malignant phenotypes and enhanced cisplatin sensitivity in vitro, while *ARMCX3* re-expression partially restored these phenotypes. Transcriptomic and molecular analyses further supported the involvement of p53-associated signaling in the biological responses following *ARMCX3* depletion.

## 5. Conclusions

In summary, the present study shows that *ARMCX3* depletion suppresses breast cancer cell proliferation, migration, invasion, and tumor growth while enhancing cisplatin sensitivity in vitro. Transcriptomic and molecular analyses showed that *ARMCX3* depletion was associated with increased p53 protein abundance and alterations in p53-associated signaling. *ARMCX3* knockdown increased p21 expression and induced G1-phase cell-cycle arrest, consistent with changes in p53-associated cell-cycle regulatory responses. In addition, *ARMCX3* depletion was associated with altered DNA damage response signaling and enhanced apoptosis following cisplatin treatment. Collectively, these findings provide new insights into the biological function of *ARMCX3* in breast cancer and support further investigation of its potential relevance to cisplatin responsiveness.

## Data Availability

The RNA-sequencing data generated in this study have been deposited in the NCBI Gene Expression Omnibus (GEO) database under accession number GSE308702. Publicly available data analyzed in this study were obtained from The Cancer Genome Atlas (TCGA), the Genotype-Tissue Expression (GTEx) project, the Clinical Proteomic Tumor Analysis Consortium (CPTAC) through the UALCAN portal, and the Human Protein Atlas (HPA). The remaining data supporting the findings of this study are available within the article and its Supplementary Materials. Additional data are available from the corresponding author upon reasonable request.

## Supplementary Materials

The following supporting information can be downloaded at: https://www.mdpi.com/article/10.3390/biology15201853/s1, Figure S1: Validation of *ARMCX3* knockdown and rescue in breast cancer cells. Figure S2: Expression changes in p53-associated genes following *ARMCX3* depletion in breast cancer cells. Figure S3: Densitometric quantification of individual p53 and DNA damage response-related proteins following cisplatin and PFT-α treatment in *ARMCX3* knockdown breast cancer cells. Figure S4: Exploratory analysis of ERK signaling following *ARMCX3* depletion in breast cancer cells. Figure S5: Exploratory analysis of migration, invasion, and EMT-associated phenotypes following trametinib treatment in *ARMCX3* knockdown breast cancer cells. Figure S6: Exploratory analysis of apoptosis and DNA damage following trametinib treatment in cisplatin-treated *ARMCX3* knockdown breast cancer cells. Figure S7: Exploratory analysis of DNA damage response signaling following trametinib treatment in cisplatin-treated *ARMCX3* knockdown breast cancer cells. Table S1: Antibody information. Table S2: Exact *p* values for the statistical comparisons presented in Figure 2. Table S3: Exact *p* values for the statistical comparisons presented in Figure 4. Table S4: Exact *p* values for the statistical comparisons presented in Figure 5. Table S5: Exact *p* values for the statistical comparisons presented in Figure 6. Table S6: Exact *p* values for the statistical comparisons presented in Figure 7. Table S7: Exact *p* values for the statistical comparisons presented in Figure 8. Table S8: Exact *p* values for the statistical comparisons presented in Figure 9. Supplementary File: Original uncropped Western blot images, raw comet assay data, and transcriptomic analysis data.
