# Supplementary material for: ARMCX3 Regulates Breast Cancer Progression and Cisplatin Responsiveness Through p53-Related Signaling
Source: Biology (Basel). 2026 Oct 15;15(20):1853. doi: 10.3390/biology15201853 (PMC13701251; doi:10.3390/biology15201853)
Supplement: Supplementary file 1 [file biology-15-01853-s001.zip › biology-4597080-supplementary.pdf]

Supplementary Materials

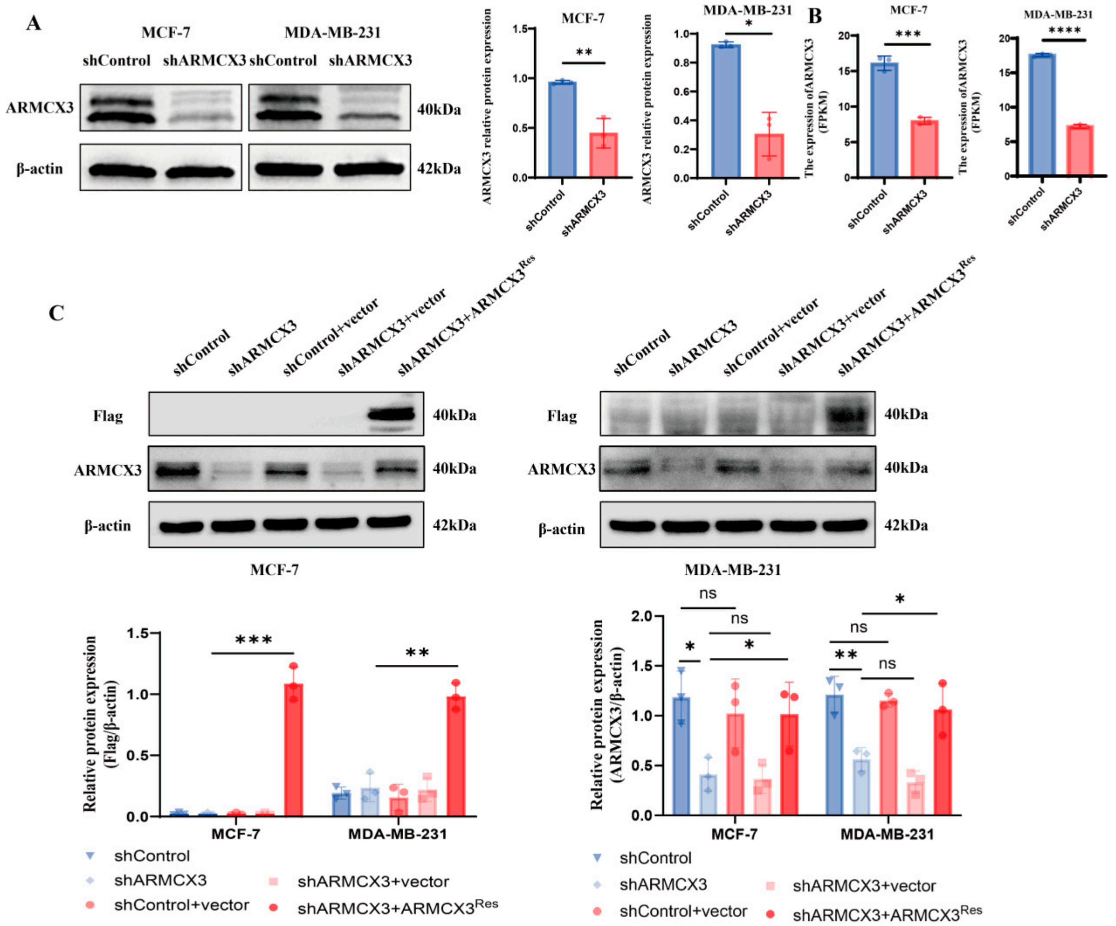

Supplementary Figure S1 **Validation of *ARMCX3* knockdown and rescue in breast cancer cells. (A)** Western blot analysis and quantification of ARMCX3 protein expression in MCF-7 and MDA-MB-231 cells stably transfected with shControl or shARMCX3. **(B)** RNA-seq analysis showing *ARMCX3* mRNA expression (FPKM) in shControl and shARMCX3 MCF-7 and MDA-MB-231 cells. **(C)** Western blot analysis and quantification of Flag-tagged and total ARMCX3 protein expression in MCF-7 and MDA-MB-231 cells transfected with shControl, shARMCX3, shControl+vector, shARMCX3 +vector, or shARMCX3+*ARMCX3*^{Res}. Data are presented as mean ± SD. ns, not significant; $^{*}P < 0.05$; $^{**}P < 0.01$; $^{***}P < 0.001$; $^{****}P < 0.0001$.

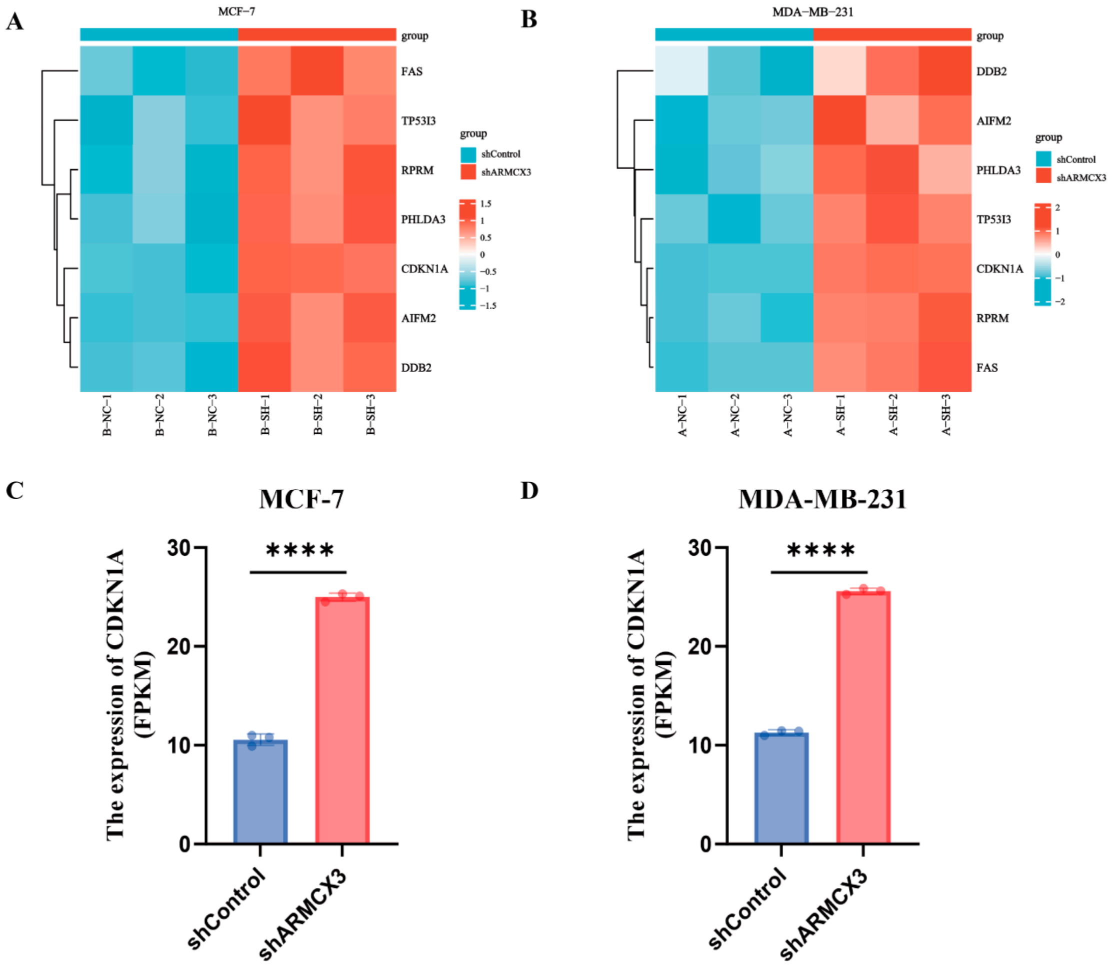

Supplementary Figure S2. **Expression changes in p53-associated genes following *ARMCX3* depletion in breast cancer cells.** (**A-B**) Heatmaps showing the expression patterns of selected p53 associated genes in shControl and shARMCX3 MCF-7 (A) and MDA-MB-231 (B) cells based on RNA-sequencing data. (**C-D**) RNA-sequencing-derived *CDKN1A* expression levels (FPKM) in shControl and shARMCX3 MCF-7 (C) and MDA-MB-231 (D) cells. Data are presented as mean ± SD. $^{****}P < 0.0001$.

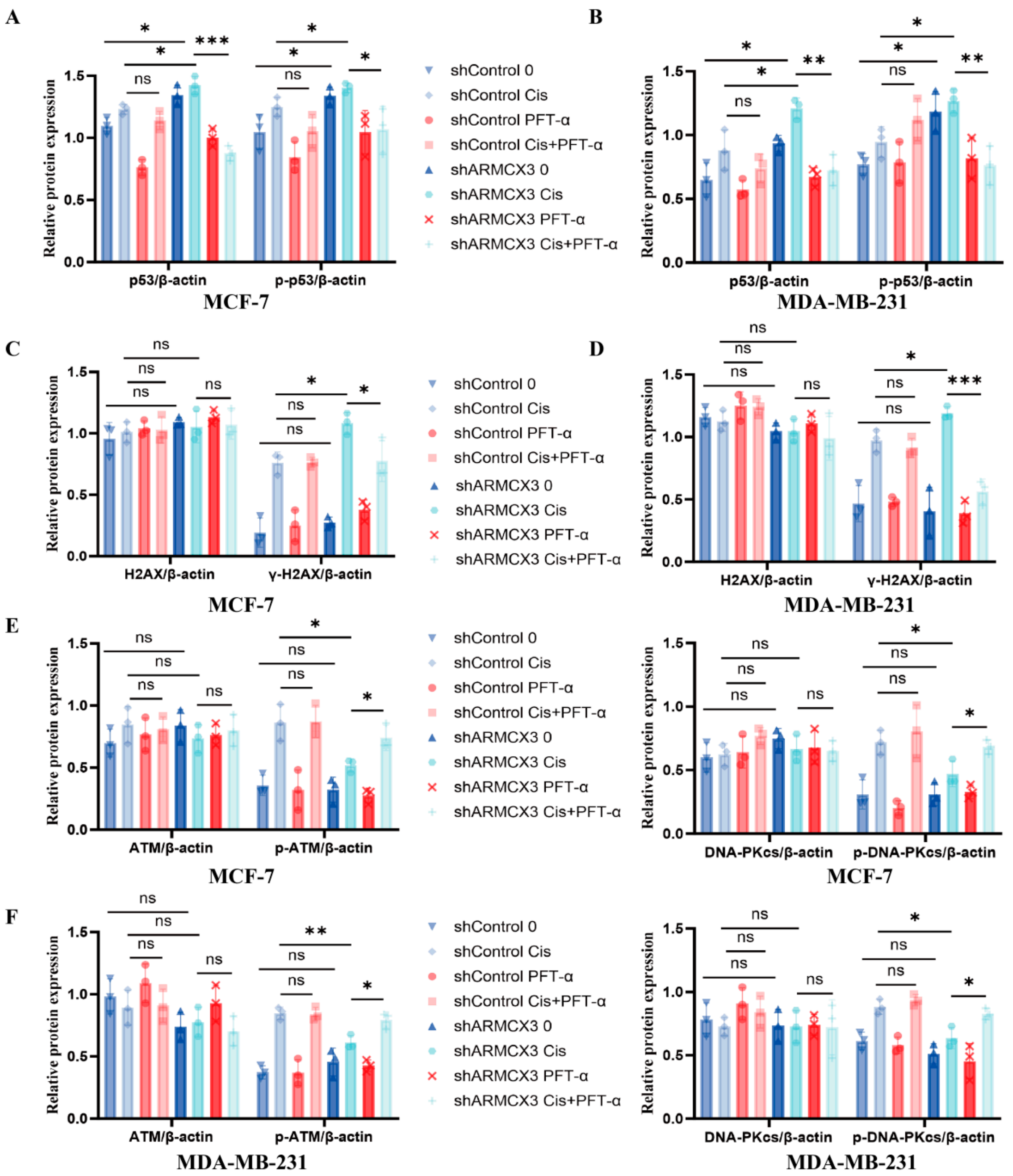

Supplementary Figure S3 **Densitometric quantification of individual p53 and DNA damage response-related proteins following cisplatin and PFT-α treatment in *ARMCX3* knockdown breast cancer cells.** (**A-B**) Quantification of p53, phosphorylated p53 (p-p53) protein levels normalized to β-actin in shControl and shARMCX3 MCF-7 (A) and MDA-MB-231 (B) cells under the indicated treatment conditions. (**C-D**) Quantification of total H2AX and γ-H2AX protein levels normalized to β-actin in MCF-7 (C) and MDA-MB-231 (D) cells. (**E-F**) Quantification of total and phosphorylated ATM and DNA-PKcs protein levels normalized to β-actin in MCF-7 (E) and MDA-MB-231 (F) cells. MCF-7 cells were treated with cisplatin (10 μM) and/or PFT-α (20 μM), whereas MDA-MB-231 cells were treated with cisplatin (20 μM) and/or PFT-α (30 μM) for 24 h. The corresponding p-p53/p53, γ-H2AX/H2AX, p-ATM/ATM, and p-DNA-PKcs/DNA-PKcs ratios are presented in Figure 8. Protein levels were normalized to β-actin. Data are presented as mean ± SD. ns, not significant; $^{*}P < 0.05$, $^{**}P < 0.01$, $^{***}P < 0.001$, $^{****}P < 0.0001$.

Supplementary Results: Exploratory analysis of ERK signaling following *ARMCX3* depletion

To explore additional signaling changes associated with *ARMCX3* depletion, we examined ERK-related signaling as a supplementary analysis. Protein interaction network analysis identified *MAPK1* and *MAPK3* among candidate genes associated with p53, while functional annotation of the RNA-sequencing dataset showed enrichment of ERK-related signaling following *ARMCX3* depletion (Figure S4A-B). *ARMCX3* knockdown increased ERK1/2 phosphorylation without markedly affecting total ERK1/2 levels, whereas *ARMCX3*^{Res} re-expression reduced ERK1/2 phosphorylation toward control levels (Figure S4C). Pharmacological inhibition of MEK with trametinib reduced ERK1/2 phosphorylation and was accompanied by decreased p53 protein abundance in both breast cancer cell models (Figure S4D-E). These observations indicate an association between ERK signaling and p53 protein abundance following *ARMCX3* depletion; however, they do not establish a direct ERK-mediated mechanism of p53 regulation.

We further examined the effects of trametinib on *ARMCX3* knockdown-associated phenotypes. Trametinib altered migration, invasion, and EMT-associated protein expression, although these effects did not consistently restore the phenotypes of *ARMCX3*-knockdown cells toward control levels (Figure S5). Therefore, these experiments do not establish ERK signaling as a necessary mediator of the migration, invasion, or EMT-related effects associated with *ARMCX3* depletion.

In cisplatin treated cells, trametinib also attenuated several changes associated with *ARMCX3* depletion, including apoptosis, γ-H2AX accumulation, comet-derived DNA damage measurements, and alterations in ATM and DNA-PKcs phosphorylation (Figure S6 and S7). Collectively, these pharmacological observations suggest that ERK signaling may be associated with some molecular responses following *ARMCX3* depletion. However, because direct genetic epistasis and biochemical analyses of ERK-mediated p53 regulation were not performed, these findings are presented as exploratory supporting observations rather than evidence of a causal *ARMCX3*-ERK-p53 signaling axis.

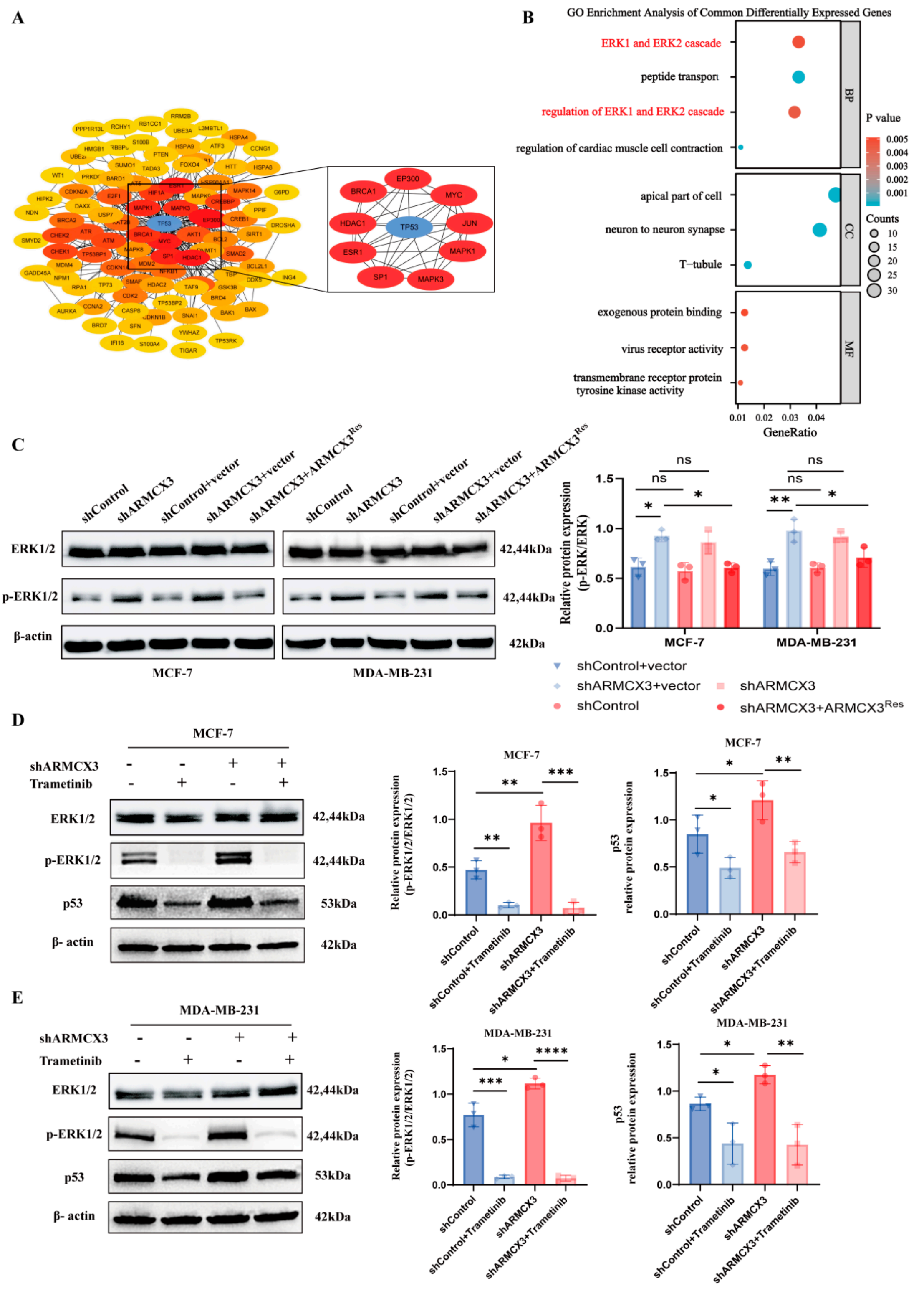

Supplementary Figure S4 **Exploratory analysis of ERK signaling following *ARMCX3* depletion in breast cancer cells.** (**A**) Protein-protein interaction (PPI) network constructed using the STRING database, identifying *TP53* as a core hub gene among differentially expressed genes. (**B**) GO enrichment analysis indicating the ERK1/2 cascade is significantly enriched. (**C**) Western blot analysis and quantification of total ERK1/2 and p-ERK1/2 in shControl, shARMCX3, shControl+vector, shARMCX3+vector, and shARMCX3 +*ARMCX3*Res MCF-7 and MDA-MB-231

cells. (**D-E**) Western blot analysis of p-ERK, total ERK and p53 protein expression in MCF-7 (D) and MDA-MB-231 (E) cells with or without trametinib intervention. MCF-7 cells were treated with 0.5 μmol/L trametinib for 48 h, whereas MDA-MB-231 cells were treated with 10 nmol/L trametinib for 48 h. Data are presented as mean ± SD. ns, not significant; $^{*}P < 0.05$, $^{**}P < 0.01$, $^{***}P < 0.001$, $^{****}P < 0.0001$.

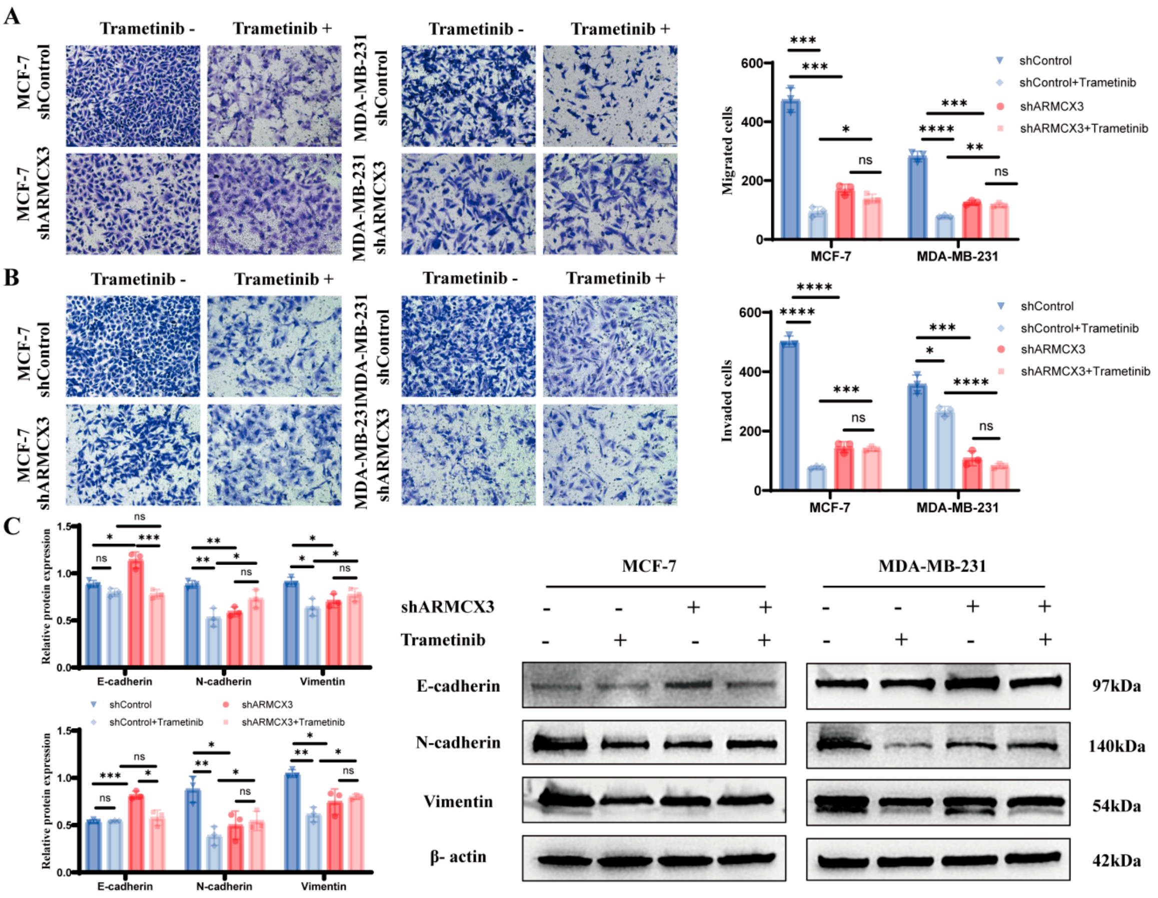

Supplementary Figure S5 **Exploratory analysis of migration, invasion, and EMT-associated phenotypes following trametinib treatment in *ARMCX3* knockdown breast cancer cells.** (**A**) Representative images and quantification of Transwell migration assays in MCF-7 and MDA-MB-231 cells. (**B**) Representative images and quantification of Transwell invasion assays. (**C**) Western blot analysis of EMT-associated protein expression in MCF-7 and MDA-MB-231 cells under the indicated treatments. MCF-7 cells were treated with 0.5 μmol/L trametinib for 48 h, whereas MDA-MB-231 cells were treated with 10 nmol/L trametinib for 48 h. Data are presented as mean ± SD. ns, not significant; $^{*}P < 0.05$, $^{**}P < 0.01$, $^{***}P < 0.001$, $^{****}P < 0.0001$.

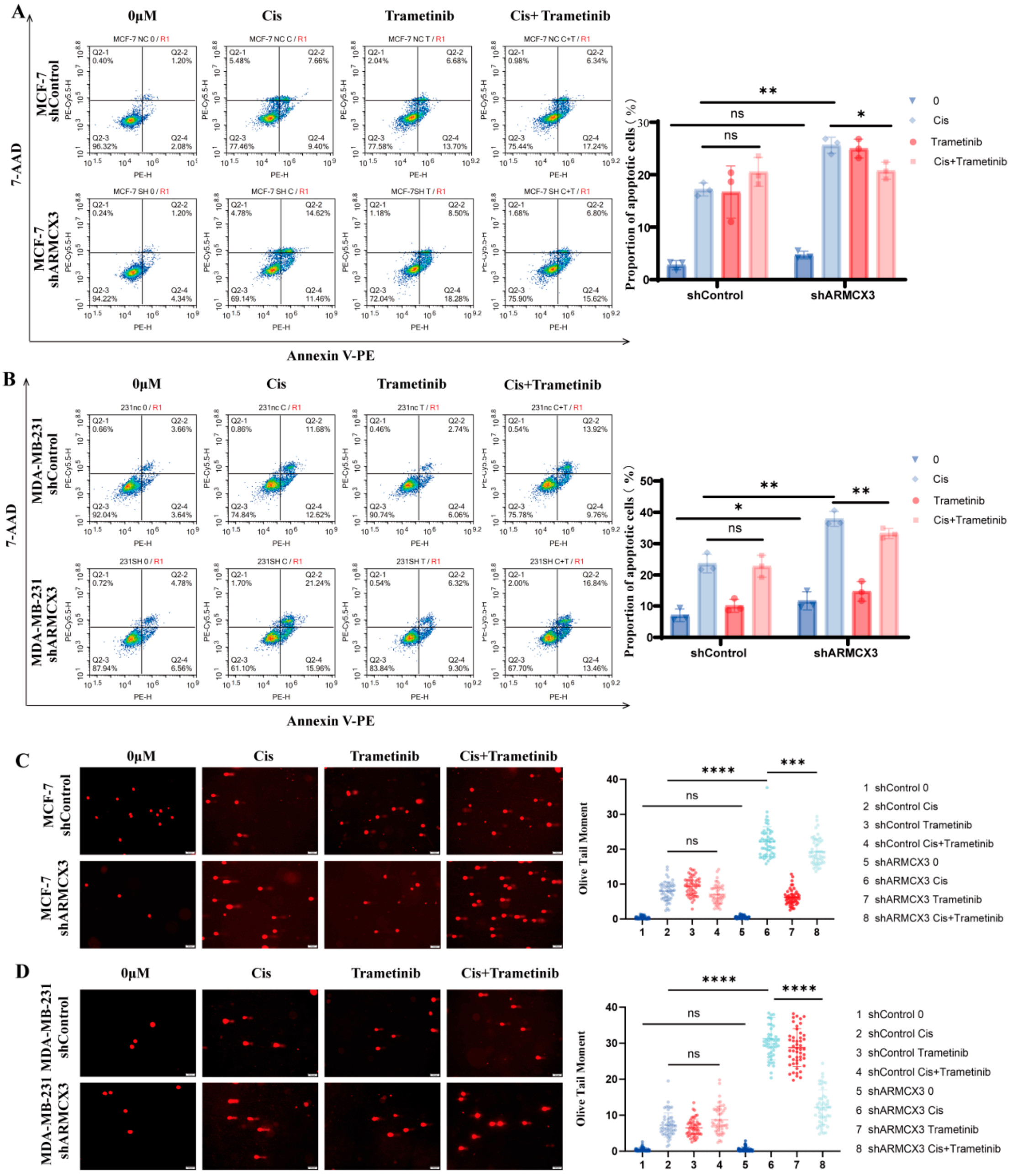

Supplementary Figure S6 **Exploratory analysis of apoptosis and DNA damage following trametinib treatment in cisplatin-treated *ARMCX3* knockdown breast cancer cells. (A–B)** Flow cytometric analysis and quantitative analysis of apoptosis in shControl and shARMCX3 MCF-7 (A) and MDA-MB-231 (B) cells following treatment with cisplatin and/or trametinib. **(C–D)** Comet assay analysis showing olive tail moment (OTM) in MCF-7 (C) and MDA-MB-231 (D) cells under the indicated treatment conditions. Scale bar, 50 µm. MCF-7 cells were treated with cisplatin (10 µM) for 24 h, while MDA-MB-231 cells were treated with cisplatin (20 µM) for 24 h. Data are presented as mean ± SD. ns, not significant; $^{*}P < 0.05$; $^{**}P < 0.01$; $^{***}P < 0.001$; $^{****}P < 0.0001$.

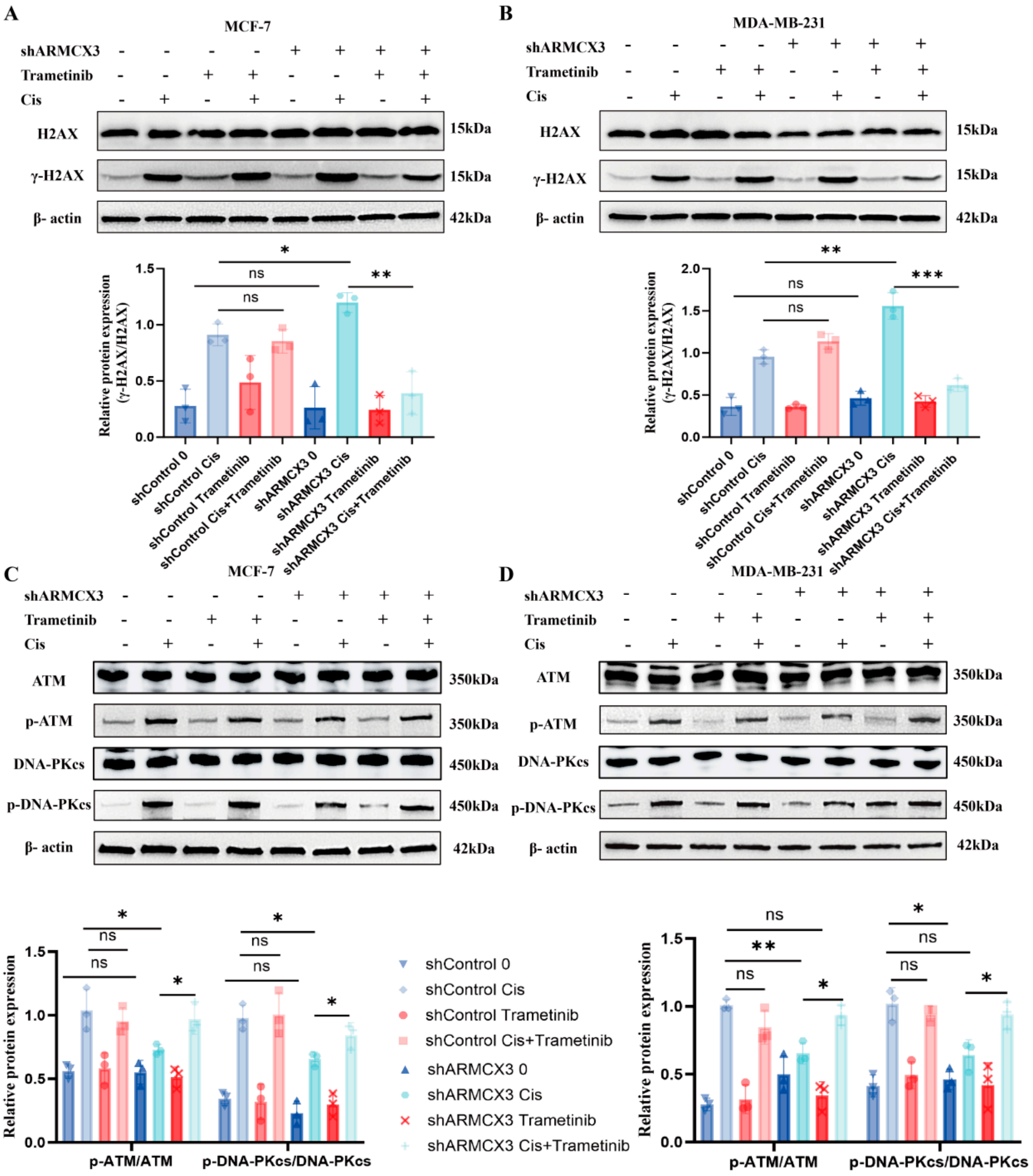

Supplementary Figure S7 **Exploratory analysis of DNA damage response signaling following trametinib treatment in cisplatin-treated *ARMCX3* knockdown breast cancer cells. (A-B)** Western blot analysis and quantification of total H2AX and γ-H2AX in shControl and shARMCX3 MCF-7 (A) and MDA-MB-231 (B) cells under the indicated treatment conditions. **(C-D)** Western blot analysis and quantification of total and phosphorylated ATM and DNA-PKcs, together with the p-ATM/ATM and p-DNA-PKcs/DNA-PKcs ratios, in MCF-7 (C) and MDA-MB-231 (D) cells under the indicated treatment conditions. MCF-7 cells were treated with cisplatin (10 μM) for 24 h, while MDA-MB-231 cells were treated with cisplatin (20 μM) for 24 h. Data are presented as mean ± SD. ns, not significant; $^{*}P < 0.05$; $^{**}P < 0.01$; $^{***}P < 0.001$; $^{****}P < 0.0001$.

Table S1. Antibody information

Antibody	Supplier
β-actin	Abways, AB003, 1:10000
Goat Anti-Rabbit IgG (HRP)	Abways, AB0101, 1:10000
ARMCX3	Proteintech, 25705-1-AP, 1:1000
p53	Abways, CY5047, 1:1000
p-p53	Abways, CY6395, 1:1000
p21 CDKN1A	Abways, CY5088, 1:1000
Flag tag	Abways, AB0030, 1:1000
CDK2	Abways, CY5020, 1:1000
Cyclin E1	Abways, CY5815, 1:1000
ERK1/2	Proteintech, 11257-1-AP, 1:2000
p-ERK1/2	Cell Signaling Technology, 4370, 1:2000
phospho-ATM	Cell Signaling Technology, 5883T, 1:1000
ATM	Cell Signaling Technology, 2873T, 1:1000
Histone H2AX	Abcam, ab229914, 1:1000
phospho-Histone H2AX	Abcam, ab81299, 1:1000
DNA-PKcs	Abcam, ab32566, 1:1000
phospho-DNA-PKcs	Abcam, ab124918, 1:5000
N-Cadherin	Abmart, T55015S, 1:1000
E-Cadherin	Abmart, TA0131S, 1:1000
Vimentin	Abmart, T55134S, 1:1000

Table S2

Figure	Comparison	*P* value
2A	shControl vs. shARMCX3	0.0018
2A	shARMCX3 vs. shARMCX3+ARMCX3^{Res}	0.006
2B	shControl vs. shARMCX3	0.0002
2B	shARMCX3 vs. shARMCX3+ARMCX3^{Res}	0.0014
2E	shControl vs. shARMCX3 (MCF-7)	0.0001
2E	shARMCX3 vs. shARMCX3+ARMCX3^{Res} (MCF-7)	0.0002
2E	shControl vs. shARMCX3 (MDA-MB-231)	<0.0001
2E	shARMCX3 vs. shARMCX3+ARMCX3^{Res} (MDA-MB-231)	<0.0001
2F	shControl vs. shARMCX3 (MCF-7)	<0.0001
2F	shARMCX3 vs. shARMCX3+ARMCX3^{Res} (MCF-7)	0.0002
2F	shControl vs. shARMCX3 (MDA-MB-231)	0.0002
2F	shARMCX3 vs. shARMCX3+ARMCX3^{Res} (MDA-MB-231)	0.0005
2G	shControl vs. shARMCX3	0.0002
2G	shARMCX3 vs. shARMCX3+ARMCX3^{Res}	0.0422
2H	shControl vs. shARMCX3	0.0008
2H	shARMCX3 vs. shARMCX3+ARMCX3^{Res}	0.0132

Table S3

Figure	Comparison	*P* value
4E	shControl vs. shARMCX3 (MCF-7)	0.0232
4E	shARMCX3 vs. shARMCX3+ARMCX3^{Res} (MCF-7)	0.0312
4E	shControl vs. shARMCX3 (MDA-MB-231)	0.0124
4E	shARMCX3 vs. shARMCX3+ARMCX3^{Res} (MDA-MB-231)	0.0164

Table S4

Figure	Comparison	*P* value
5A	shControl vs. shARMCX3	<0.0001
5A	shARMCX3 vs. shARMCX3+PFT-α	0.0017
5B	shControl vs. shARMCX3	<0.0001
5B	shARMCX3 vs. shARMCX3+PFT-α	0.0012
5C	shControl vs. shARMCX3 (MCF-7)	0.0004
5C	shARMCX3 vs. shARMCX3+PFT-α (MCF-7)	0.0054
5C	shControl vs. shARMCX3 (MDA-MB-231)	0.0014
5C	shARMCX3 vs. shARMCX3+PFT-α (MDA-MB-231)	0.0460
5D	shControl vs. shARMCX3 (MCF-7)	0.0011
5D	shARMCX3 vs. shARMCX3+PFT-α (MCF-7)	0.0014
5D	shControl vs. shARMCX3 (MDA-MB-231)	0.0081
5D	shARMCX3 vs. shARMCX3+PFT-α (MDA-MB-231)	0.0244
5E	shControl vs. shControl+PFT-α (MCF-7, p53)	0.0029
5E	shControl vs. shARMCX3 (MCF-7, p53)	0.0219
5E	shARMCX3 vs. shARMCX3+PFT-α (MCF-7, p53)	0.0003
5E	shControl vs. shARMCX3 (MCF-7, E-cadherin)	0.0071
5E	shARMCX3 vs. shARMCX3+PFT-α (MCF-7, E-cadherin)	0.0280
5E	shControl vs. shARMCX3 (MCF-7, N-cadherin)	0.0060
5E	shARMCX3 vs. shARMCX3+PFT-α (MCF-7, N-cadherin)	0.0127
5E	shControl vs. shARMCX3 (MCF-7, vimentin)	0.0092
5E	shARMCX3 vs. shARMCX3+PFT-α (MCF-7, vimentin)	0.0222
5E	shControl vs. shControl+PFT-α (MDA-MB-231, p53)	0.0157
5E	shControl vs. shARMCX3 (MDA-MB-231, p53)	0.0102
5E	shARMCX3 vs. shARMCX3+PFT-α (MDA-MB-231, p53)	0.0036
5E	shControl vs. shARMCX3 (MDA-MB-231, E-cadherin)	0.0044
5E	shARMCX3 vs. shARMCX3+PFT-α (MDA-MB-231, E-cadherin)	0.0015
5E	shControl vs. shARMCX3 (MDA-MB-231, N-cadherin)	0.0007
5E	shARMCX3 vs. shARMCX3+PFT-α (MDA-MB-231, N-cadherin)	0.0309
5E	shControl vs. shARMCX3 (MDA-MB-231, vimentin)	0.0039
5E	shARMCX3 vs. shARMCX3+PFT-α (MDA-MB-231, vimentin)	0.0281

Table S5

Figure	Comparison	*P* value
6A	shControl vs. shARMCX3 (G1)	0.0004
6A	shARMCX3 vs. shARMCX3+PFT-α (G1)	0.0005
6A	shControl vs. shARMCX3 (S)	0.0004
6A	shARMCX3 vs. shARMCX3+PFT-α (S)	0.0003
6B	shControl vs. shARMCX3 (G1)	0.0014
6B	shARMCX3 vs. shARMCX3+PFT-α (G1)	0.0160
6B	shControl vs. shARMCX3 (S)	0.0060
6B	shARMCX3 vs. shARMCX3+PFT-α (S)	0.0002
6C	shControl vs. shControl+PFT-α (MCF-7, p53)	0.0427
6C	shControl vs. shARMCX3 (MCF-7, p53)	0.0080
6C	shARMCX3 vs. shARMCX3+PFT-α (MCF-7, p53)	0.0039
6C	shControl vs. shControl+PFT-α (MCF-7, p-p53)	0.0298
6C	shControl vs. shARMCX3 (MCF-7, p-p53)	0.0268
6C	shARMCX3 vs. shARMCX3+PFT-α (MCF-7, p-p53)	0.002
6C	shControl vs. shControl+PFT-α (MCF-7, p21)	0.0156
6C	shControl vs. shARMCX3 (MCF-7, p21)	0.0047
6C	shARMCX3 vs. shARMCX3+PFT-α (MCF-7, p21)	<0.0001
6C	shControl vs. shARMCX3 (MCF-7, CDK2)	0.0082
6C	shARMCX3 vs. shARMCX3+PFT-α (MCF-7, CDK2)	0.0427
6C	shControl vs. shARMCX3 (MCF-7, Cyclin E1)	0.0053
6C	shARMCX3 vs. shARMCX3+PFT-α (MCF-7, Cyclin E1)	0.0205
6D	shControl vs. shControl+PFT-α (MDA-MB-231, p53)	0.0062
6D	shControl vs. shARMCX3 (MDA-MB-231, p53)	0.0043
6D	shARMCX3 vs. shARMCX3+PFT-α (MDA-MB-231, p53)	0.0080
6D	shControl vs. shControl+PFT-α (MDA-MB-231, p-p53)	0.0045
6D	shControl vs. shARMCX3 (MDA-MB-231, p-p53)	0.0340
6D	shARMCX3 vs. shARMCX3+PFT-α (MDA-MB-231, p-p53)	0.0006
6D	shControl vs. shControl+PFT-α (MDA-MB-231, p21)	0.0234
6D	shControl vs. shARMCX3 (MDA-MB-231, p21)	0.0021
6D	shARMCX3 vs. shARMCX3+PFT-α (MDA-MB-231, p21)	0.0111
6D	shControl vs. shARMCX3 (MDA-MB-231, CDK2)	0.0087
6D	shARMCX3 vs. shARMCX3+PFT-α (MDA-MB-231, CDK2)	0.0310
6D	shControl vs. shARMCX3 (MDA-MB-231, Cyclin E1)	0.0412
6D	shARMCX3 vs. shARMCX3+PFT-α (MDA-MB-231, Cyclin E1)	0.0382

Table S6

Figure	Comparison	*P* value
7A	shControl Cis vs. shARMCX3 Cis	0.0236
7A	shARMCX3 Cis vs. shARMCX3 Cis+PFT-α	0.0475
7B	shControl 0μM vs. shARMCX3 0μM	0.0025
7B	shControl Cis vs. shARMCX3 Cis	0.0001
7B	shARMCX3 Cis vs. shARMCX3 Cis+PFT-α	0.0074
7C	shControl Cis vs. shARMCX3 Cis	<0.0001
7C	shARMCX3 Cis vs. shARMCX3 Cis+PFT-α	<0.0001
7D	shControl Cis vs. shARMCX3 Cis	<0.0001
7D	shARMCX3 Cis vs. shARMCX3 Cis+PFT-α	<0.0001

Table S7

Figure	Comparison	*P* value
8C	shControl Cis vs. shARMCX3 Cis (γ-H2AX/ H2AX)	0.0484
8C	shARMCX3 Cis vs. shARMCX3 Cis+PFT-α (γ-H2AX/ H2AX)	0.0474
8D	shControl Cis vs. shARMCX3 Cis (γ-H2AX/ H2AX)	0.0112
8D	shARMCX3 Cis vs. shARMCX3 Cis+PFT-α (γ-H2AX/ H2AX)	0.0042
8E	shControl Cis vs. shARMCX3 Cis (p-ATM/ATM)	0.0267
8E	shARMCX3 Cis vs. shARMCX3 Cis+PFT-α (p-ATM/ATM)	0.0249
8E	shControl Cis vs. shARMCX3 Cis (p-DNA-PKcs/DNA-PKcs)	0.0138
8E	shARMCX3 Cis vs. shARMCX3 Cis+PFT-α (p-DNA-PKcs/DNA-PKcs)	0.0142
8F	shControl Cis vs. shARMCX3 Cis (p-ATM/ATM)	0.0304
8F	shARMCX3 Cis vs. shARMCX3 Cis+PFT-α (p-ATM/ATM)	0.0481
8F	shControl Cis vs. shARMCX3 Cis (p-DNA-PKcs/DNA-PKcs)	0.0044
8F	shARMCX3 Cis vs. shARMCX3 Cis+PFT-α (p-DNA-PKcs/DNA-PKcs)	0.0017

Table S8

Figure	Comparison	*P* value
9B	shControl vs. shARMCX3	<0.0001
9B	shARMCX3 vs. shARMCX3+PFT-α	0.0023
9C	shControl vs. shARMCX3	<0.0001
9C	shARMCX3 vs. shARMCX3+PFT-α	<0.0001
9C	shControl+PFT-α vs. shARMCX3+PFT-α	<0.0001
9E	shControl vs. shARMCX3	0.0049
9E	shARMCX3 vs. shARMCX3+PFT-α	0.0489
9F	shControl vs. shARMCX3	<0.0001
9F	shARMCX3 vs. shARMCX3+PFT-α	<0.0001
9F	shControl+PFT-α vs. shARMCX3+PFT-α	0.0003
9G	shControl vs. shARMCX3(Ki67)	<0.0001
9G	shARMCX3 vs. shARMCX3+PFT-α(Ki67)	0.0002
9G	shControl vs. shARMCX3(cleaved-Caspase3)	<0.0001
9G	shARMCX3 vs. shARMCX3+PFT-α(cleaved-Caspase3)	<0.0001
9H	shControl vs. shARMCX3(Ki67)	<0.0001
9H	shARMCX3 vs. shARMCX3+PFT-α(Ki67)	0.0003
9H	shControl vs. shARMCX3(cleaved-Caspase3)	0.0029
9H	shARMCX3 vs. shARMCX3+PFT-α(cleaved-Caspase3)	0.0024
